# Agonist-controlled competition of RAR and VDR nuclear receptors for heterodimerization with RXR is manifested in their DNA binding

**DOI:** 10.1016/j.jbc.2023.102896

**Published:** 2023-01-11

**Authors:** Bálint Rehó, Lina Fadel, Peter Brazda, Anass Benziane, Éva Hegedüs, Pialy Sen, Theodorus W.J. Gadella, Katalin Tóth, László Nagy, György Vámosi

**Affiliations:** 1 Department of Biophysics and Cell Biology, Faculty of Medicine, Doctoral School of Molecular Medicine, University of Debrecen, Debrecen, Hungary; 2 Department of Biochemistry and Molecular Biology, Faculty of Medicine, University of Debrecen, Debrecen, Hungary; 3 Princess Maxima Centre for Pediatric Oncology, Utrecht, the Netherlands; 4 Section of Molecular Cytology and van Leeuwenhoek Centre for Advanced Microscopy (LCAM), Swammerdam Institute for Life Sciences, University of Amsterdam, Amsterdam, The Netherlands; 5 Department of Medicine and Biological Chemistry, Johns Hopkins University School of Medicine, Institute for Fundamental Biomedical Research, Johns Hopkins All Children's Hospital, Saint Petersburg, Florida, USA

**Keywords:** nuclear receptors, RAR, VDR, RXR, competition, DNA binding, fluorescence correlation spectroscopy, FRET, FCS, ACF, autocorrelation function, AM580, RAR-specific agonist, DBD, DNA-binding domain, EGFP, enhanced green fluorescent protein, FCCS, fluorescence cross-correlation spectroscopy, FCS, fluorescence correlation spectroscopy, FLIM, fluorescence lifetime imaging microscopy, FP, fluorescent protein, FRET, Förster resonance energy transfer, HEK293, human embryonic kidney adherent cells, IRF, instrument response function, LBD, ligand-binding domain, LG268, RXR-specific agonist, LXR, liver X receptor, NR, nuclear receptor, PPARγ, peroxisome proliferator-activated receptor gamma, RAR, retinoic acid receptor, RXR, retinoid X receptor, TagBFP, blue fluorescent protein, TZD, thiazolidinedione, VDR, vitamin D receptor

## Abstract

We found previously that nuclear receptors (NRs) compete for heterodimerization with their common partner, retinoid X receptor (RXR), in a ligand-dependent manner. To investigate potential competition in their DNA binding, we monitored the mobility of retinoic acid receptor (RAR) and vitamin D receptor (VDR) in live cells by fluorescence correlation spectroscopy. First, specific agonist treatment and RXR coexpression additively increased RAR DNA binding, while both agonist and RXR were required for increased VDR DNA binding, indicating weaker DNA binding of the VDR/RXR dimer. Second, coexpression of RAR, VDR, and RXR resulted in competition for DNA binding. Without ligand, VDR reduced the DNA-bound fraction of RAR and vice versa, *i.e.*, a fraction of RXR molecules was occupied by the competing partner. The DNA-bound fraction of either RAR or VDR was enhanced by its own and diminished by the competing NR’s agonist. When treated with both ligands, the DNA-bound fraction of RAR increased as much as due to its own agonist, whereas that of VDR increased less. RXR agonist also increased DNA binding of RAR at the expense of VDR. In summary, competition between RAR and VDR for RXR is also manifested in their DNA binding in an agonist-dependent manner: RAR dominates over VDR in the absence of agonist or with both agonists present. Thus, side effects of NR-ligand-based (retinoids, thiazolidinediones) therapies may be ameliorated by other NR ligands and be at least partly explained by reduced DNA binding due to competition. Our results also complement the model of NR action by involving competition both for RXR and for DNA sites.

Nuclear receptors (NRs) are transcription factors that regulate gene expression in a ligand-dependent manner. A subgroup of NRs, retinoic acid receptors (RARs), and vitamin D receptor (VDR) play a pivotal role in cell functions like cell growth, development, and cell death (1, 2, 3). Retinoid X receptors (RXRs) are crucial in the activation of NRs because they are obligatory heterodimerizing partners for various NRs including RARs and VDR (4).

The ligand-dependent activation of NRs is described by the molecular switch model (5). According to this model, NRs are bound to the chromatin at their specific binding sites called hormone (or NR) response elements and are associated with corepressor complexes. In the presence of agonist ligands, a conformational change occurs, the corepressor complexes are released, and coactivator complexes are bound, thereby activating transcription. Recently, several studies suggested that this system has a more dynamic nature than outlined above (6, 7, 8, 9, 10, 11, 12, 13, 14).

In our previous biophysical studies, we have carried out confocal fluorescence correlation spectroscopy (FCS) measurements to determine the diffusion properties of receptor complexes. We have shown that RARα and RXRα (referred to from hereon as RAR and RXR) have two distinct subpopulations present in the nucleus in the absence of ligands: a fast population corresponding to monomeric or small oligomeric forms that diffuse freely or are bound transiently with short residence times on the DNA and a slow population corresponding to complexes interacting with DNA more stably (6, 7, 14). Ligand binding shifted the balance between the two populations toward the slower one. In these measurements, only one type of nuclear receptor (RAR or RXR) was overexpressed without sufficient amounts of its heterodimerizing partner being present. In the present study, we investigated how coexpression of RXR influences the DNA binding of two of its partner receptors, RAR and VDR.

Recently, we have measured Förster resonance energy transfer and fluorescence cross-correlation spectroscopy implemented in a selected plane illumination microscope (SPIM-FRET-FCCS) to map protein–protein and protein–DNA interactions simultaneously in a selected plane of a cell. We showed that both RAR-RXR heterodimerization and DNA binding were enhanced upon RAR or RXR agonist treatment (7).

Retinoid therapy is effectively used in several dermatological conditions such as acne and psoriasis. It is also approved for the treatment of cancer, mainly acute promyelocytic leucaemia (15, 16). Vitamin D plays a role in calcium homeostasis and also acts as an immune modulator; its beneficial effects have been proven in the auxiliary treatment of COVID-19 infection (17, 18). While NRs are frequent drug targets in numerous diseases, a long-term systemic application of specific NR agonists has been associated with several side effects, which could limit their application; *e.g.*, retinoid treatment caused symptoms of vitamin D deficiency (3). This could, at least in part, be due to competition for heterodimerizing with RXR and for response elements on the DNA. Rosiglitazone treatment was found to be associated with macular edema (19), which may also be caused by disruption of retinoic acid signaling (20).

Previously, we have reported competition between NRs for heterodimerization with RXR using a nuclear translocation assay that detected protein–protein interactions between NRs in HEK cells expressing RXR and two partner NRs (21). The binding affinity of RAR to RXR was larger than that of VDR in the absence of ligand, whereas in the presence of specific agonist, always the liganded partner dominated.

Here, we further addressed the question whether the competition detected between NRs at the level of protein–protein interactions is also manifested at the level of DNA binding. To this end, we applied FCS in our previously used model system to study competition between RAR and VDR in cells expressing a limiting pool of RXR, in the presence and absence of agonists.

Based on our new results, we refined the molecular switch model of NR activation with new aspects: coexpression of RXR with its NR partner promotes their DNA binding, and competition with another NR for heterodimerization with RXR, directed by a hierarchy of affinities toward RXR and the presence of agonists, is manifested also in their DNA binding.

## Results

### Characterization of diffusing NR populations in cotransfected cells and model selection for evaluation of FCS measurements

**Figure 1:**
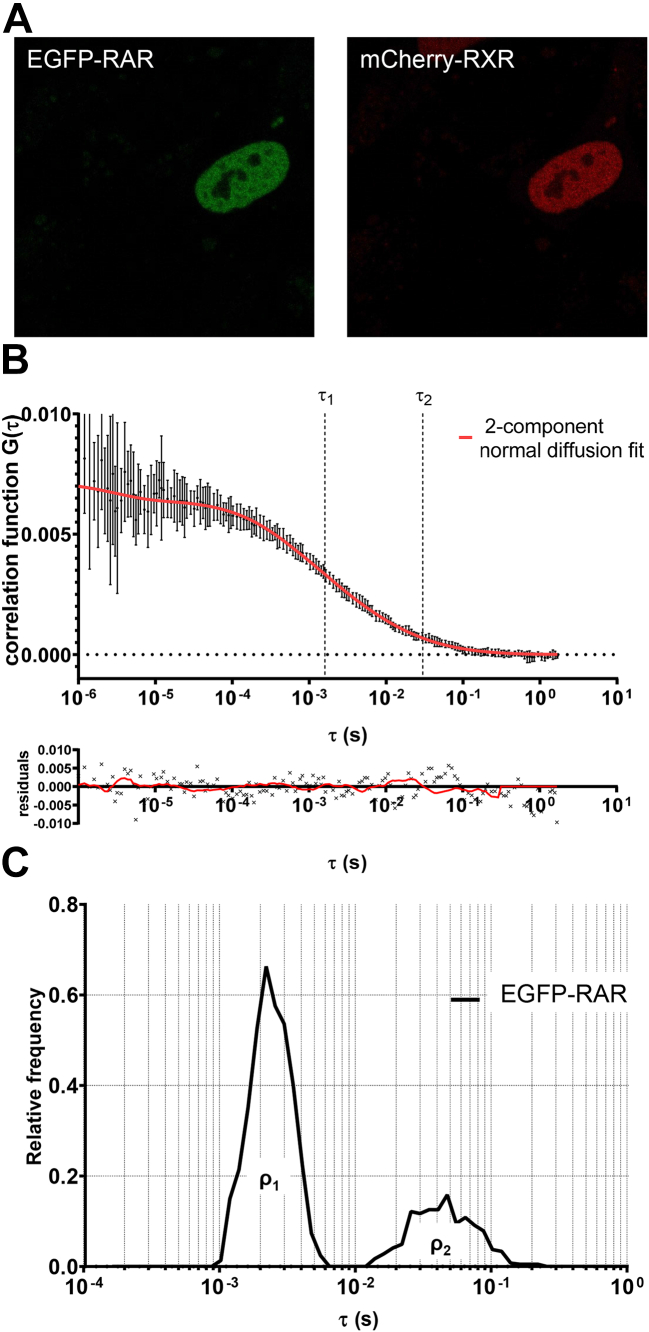
**Expression and mobility of RAR in live cells.***A*, representative confocal image of EGFP-RAR and mCherry-RXR coexpressed in HEK293 cells (image size: 71 × 71 μm). *B*, autocorrelation function determined by fluorescence correlation spectroscopy at a selected point in the nucleus and fit with a model function assuming two diffusing components of EGFP-RAR, as well as triplet state formation and blinking of EGFP. The diffusion times of the fast and slow components, τ_1_ and τ_2_, are indicated. *C*, distribution of the fast and slow diffusion times, the areas indicating their fractions ρ_1_ and ρ_2_. Further autocorrelation curves and fit models are shown in Fig. S2.

To study the DNA binding of NRs, we measured their mobility by FCS, a fluorescence microscopy technique with single molecule sensitivity. In FCS, the fluorescence intensity fluctuating due to diffusion of labeled molecules in and out of a small confocal volume (and due to photophysical processes) is recorded (22, 23). From this signal an autocorrelation function (ACF) is extracted, which reflects the photophysical and diffusion properties of the molecules. By fitting the ACF curves to model functions, the diffusion times (the average dwell times in the confocal volume), the resulting diffusion coefficients, and the fractions of molecular subpopulations can be determined (24). To minimize photobleaching, one of the most photostable fluorescent proteins, enhanced green fluorescent protein (EGFP), was used to label the studied NR. RXR and the competing partner receptor were tagged with mCherry or TagBFP (blue fluorescent protein) to visualize their distribution and estimate their relative expression levels (Fig. 1*A*).

For FCS measurements we expressed fluorescent protein-tagged NRs in HEK293 human embryonic kidney cells used previously to study competition of NRs for RXR (21); the relatively low endogenous expression of NRs in these cells makes it a good model system. In coexpression experiments, cells with equal expression levels of EGFP-, mCherry- and TagBFP-tagged NRs were selected. To achieve this, green-to-red or blue-to-green fluorescence intensity ratios of fluorescent protein (FP)-NRs were compared with those of our standards: EGFP-mCherry and TagBFP-EGFP (or EGFP-TagBFP) fusion proteins expressing the FPs at a 1:1 ratio as described earlier (21, 25, 26). The ratio of transfected, FP-tagged to endogenous, nontagged NRs in cells used for FCS was also assessed by using a combination of Western blot and confocal microscopy, yielding 0.21 for EGFP-RAR/RAR, 0.27 for EGFP-VDR/VDR, and 1.19 for mCherry-RXR/RXR (for blots, see Fig. S1; for the procedure, see Experimental procedures).

NRs diffuse in the nucleus and bind transiently or stably to their response elements on the DNA; this dynamic behavior is reflected by the ACF (Fig. 1*B*). First, we defined the minimal model accounting for the measured ACF curves. We tested four models: one or two components with normal or anomalous diffusion; in all models, triplet formation and EGFP blinking was also considered. A single component did not fit the curves sufficiently (see Fig. S2, *A* and *B*). The two-component normal diffusion model gave a tight fit to the data, which was not significantly improved further by the two-component anomalous diffusion model (Fig. S2, *C* and *D*). Based on these findings and the goodness of fit analyses (Table S1), for all measurements on full-length NRs we used the two-component normal diffusion model. Figure 1*B* shows an ACF curve of EGFP-RAR coexpressed with mCherry-RXR. The two components have significantly different diffusion times.

Previously we calculated the apparent molecular masses of these components by comparing their diffusion times with that of the EGFP monomer (14). The fast one had an apparent mass of 5 to 10 times, whereas the slow one ∼10^6^ times the molecular mass of the EGFP-RAR. Thus, the fast component (*τ*_*1*_) around 2 ms likely corresponds to a mixture of freely diffusing small complexes and transiently DNA-bound receptors scanning the DNA with short residence times. The slow one (*τ*_*2*_) is characterized by a diffusion time of ∼50 ms. Thus, the slow component may not be a freely diffusing complex but represents NRs bound to DNA more stably with longer residence times, likely at NR response elements. Recently, we have carried out FRAP experiments with EGFP-RAR (27). FRAP recovery curves recorded over 40 s could be fitted with a slow and a fast component both in the absence and in the presence of ligand, without any significant immobile fraction that would indicate stable binding on the time scale of the experiment. Thus, even the more stably bound slow fraction is only transiently attached to DNA.

The histogram in Figure 1*C* displays the distribution of the fast and slow diffusion times of EGFP-RAR. Such a histogram demonstrates the distribution of the diffusion times of the components, and the areas below the peaks are proportional to the molecular fractions. Similar histograms for VDR are shown in Fig. S3. The two components appear as distinct peaks, the widths characterizing the subcellular and cell-to-cell variation of these parameters. From these distributions, box-and-whiskers plots of the diffusion constants (*D*_*1*_, *D*_*2*_) and the fractions of the slow component (*ρ*_*2*_) were created for easier comparison of different samples (see later sections). The diffusion constant is inversely proportional to the diffusion time (dwell time); see Equation 5.

**Figure 2:**
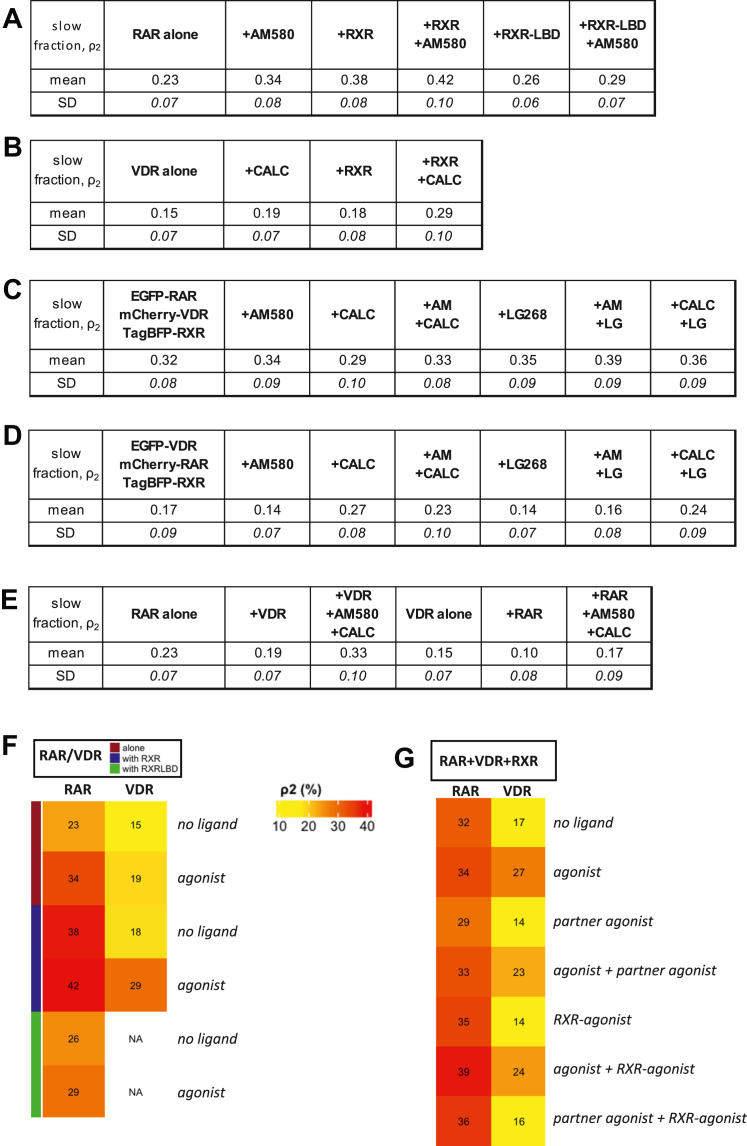
**Fractions of the slow component (ρ**_**2**_**) from fluorescence correlation spectroscopy measurements.** Slow fraction of (*A*) EGFP-RAR expressed alone or cotransfected with mCherry-RXR or mCherry-RXR-LBD in live HEK293 cells; (*B*) VDR in live HEK293 cells; (*C*) EGFP-RAR when cotransfected with mCherry-VDR, measured in stably TagBFP-RXR-expressing HEK293^BFP-RXR^ cells; (*D*) EGFP-VDR when cotransfected with mCherry-RAR, measured in stably TagBFP-RXR-expressing HEK293^BFP-RXR^ cells; (*E*) EGFP-RAR when cotransfected with mCherry-VDR, and EGFP-VDR when cotransfected with mCherry-RAR measured in live HEK293 cells. *F*, slow fractions shown as heat maps for easier overview of data in parts *A* and *B*; numbers indicate fractions as percentages; (*G*) heat maps of data in parts *C* and *D*.

Subsequently, we monitored the amount of stably DNA-bound EGFP-NR by the *ρ*_*2*_ parameter (numerical values are summarized in Fig. 2, *A*–*E* and also presented as heat maps in Fig. 2, *F* and *G* for the different experiments) and the mobility of this component by the *D*_*2*_ diffusion coefficient.

### Agonist treatment and coexpression of RXR additively increase chromatin binding of RAR

**Figure 3:**
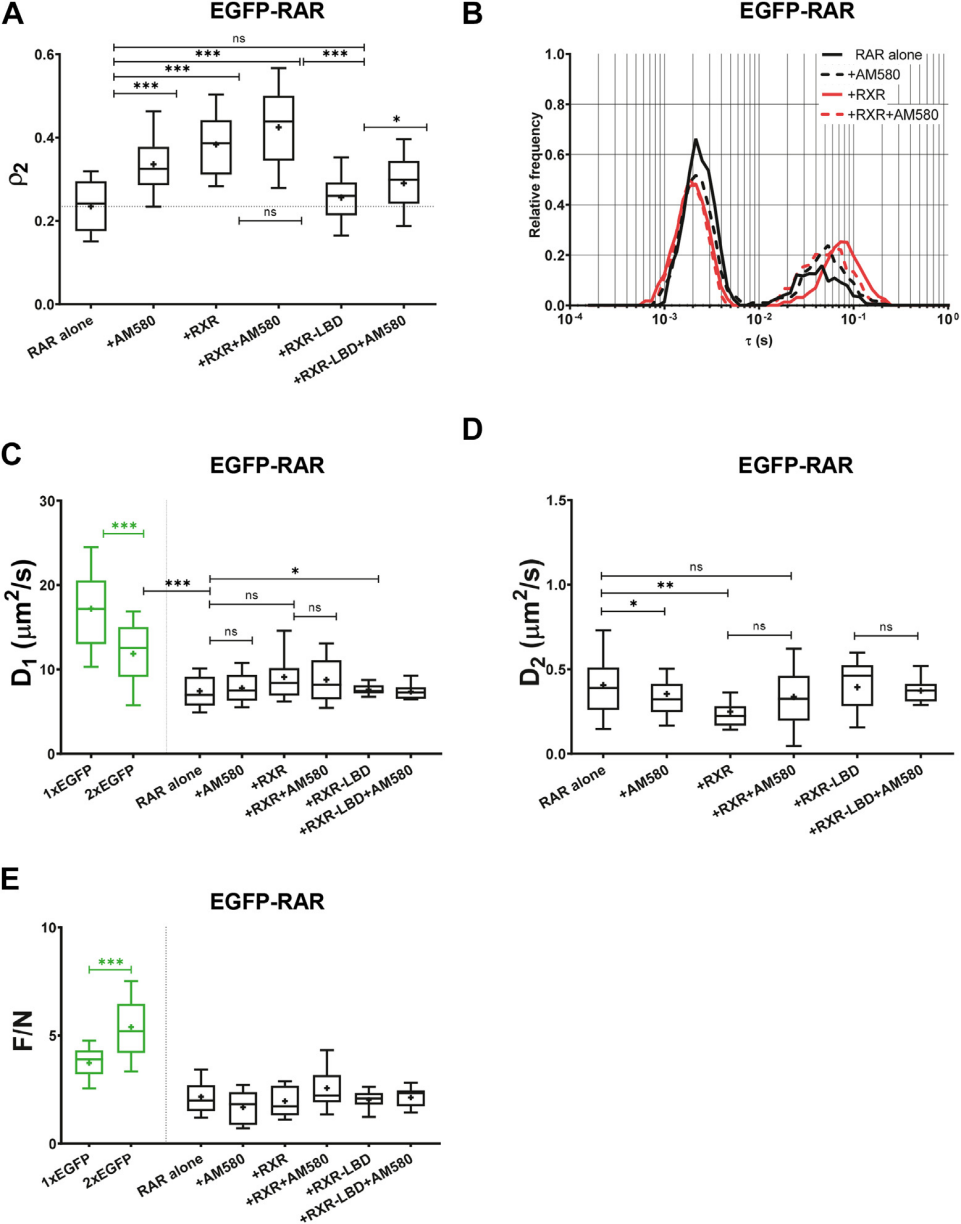
**Mobility parameters of EGFP-RAR expressed alone or cotransfected with mCherry-RXR or mCherry-RXR-LBD in live HEK293 cells with or without RAR agonist treatment.***A*, fraction of the slow component of EGFP-RAR. Cells were treated with 100 nM RAR agonist for 20 min (+AM580) and/or cotransfected with mCherry-RXR (+RXR) or its ligand-binding domain mCherry-RXR-LBD (+RXR-LBD) lacking direct DNA-binding capacity. *B*, distribution of the fast and slow diffusion times of EGFP-RAR, areas under the curves indicating their fractions. *C*, diffusion coefficient of the EGFP-RAR fast component. For comparison, the diffusion coefficients of monomeric and dimeric EGFP (1xEGFP, 2xEGFP) are shown. *D*, diffusion coefficient of the EGFP-RAR slow component. *E*, molecular brightness of EGFP-RAR. Brightness values of monomeric and dimeric EGFP are also shown. Numbers of measurements are shown in Table S2. Boxes mark the 25th and 75th percentiles while whiskers the 10th and 90th percentile values. The *horizontal line* in the box represents the median. Averages are marked by “+.” To compare averages of ρ_2_ and F/N values, *t* tests were performed; the distributions of diffusion times weighted by their fractions were compared by an F-test. ∗*p* < 0.05; ∗∗*p* < 0.01; ∗∗∗*p* < 0.001; ns, not significant.

We carried out FCS measurements on EGFP-RAR expressed alone or coexpressed with RXR in HEK293 cells, in the absence or in the presence of RAR agonist. The average value of the *ρ*_*2*_ slow fraction of EGFP-RAR expressed alone was ∼22% (Figs. 2, *A* and *F* and 3*A*). Treatment with a saturating concentration of the synthetic agonist, 10^−7^ M AM580, increased *ρ*_*2*_ to ∼34% (Fig. 3*A*) and reduced its diffusion constant *D*_*2*_ (Fig. 3*D*), likely reflecting an increase of its residence time on DNA. This suggests that agonist binding induces a conformational change enhancing the DNA binding of RAR itself. Addition of RXR alone increased the slow fraction and decreased *D*_*2*_ to an even greater extent. The ratio of cotransfected mCherry-RXR to endogenous RXR is 1.19; *i.e.*, transfection increases the RXR pool by ∼120% enhancing the fraction of RAR/RXR heterodimers. RXR coexpression and ligand treatment applied together had an additive effect on the DNA binding of RAR further increasing the DNA-bound fraction (Fig. 3*A*). The mobility of the fast component, corresponding to less stably bound or freely diffusing receptors, was not affected by any of the treatments as reflected by the diffusion constant *D*_*1*_ (Fig. 3*C*). Figure 3*B* summarizes these changes: the peak of the slow component (on the right) is shifted to longer diffusion times, and its area is increased upon the applied treatments. We got a similar behavior of the slow fraction of RAR in response to AM580 treatment and RXR cotransfection in HeLa cells as well (Fig. S4), in which the endogenous transcript ratios of RAR/RXR and VDR/RXR are 4 to 5 times lower as compared with HEK293 (according to the Human Protein Atlas (28) (http://www.proteinatlas.org)).

To investigate the mechanism by which coexpression of RXR enhances RAR DNA binding, we also used a non-DNA-binding variant of RXR having only the ligand-binding domain (LBD), which is also responsible for dimerization. Contrary to full-length RXR, cotransfection of RXR-LBD to full-length RAR did not increase the slow fraction in the absence of the ligand, and a minor increase happened in the presence of agonist (first *versus* fifth columns and fifth *versus* sixth columns in Fig. 3*A*). Thus, dimerization with RXR enhances chromatin binding of RAR only if RXR itself has DNA binding capability. The mobility of the fast component was not influenced to an observable extent by the presence of RXR-LBD either (Fig. 3*C*).

The molecular brightness of the diffusing particles is proportional to the number of fluorophores moving together in a complex, *i.e.*, it reflects potential aggregation. Figure 3*E* shows that neither the presence of RXR nor agonist treatment influenced the number of RAR molecules in the complex dramatically. The somewhat smaller brightness of EGFP-RAR as compared with monomeric EGFP may be due to modulation of the fluorescence quantum yield by the linker peptide, the fusion protein, or its interacting partners (29).

### VDR mobility decreases only when both agonist and RXR are present

**Figure 4:**
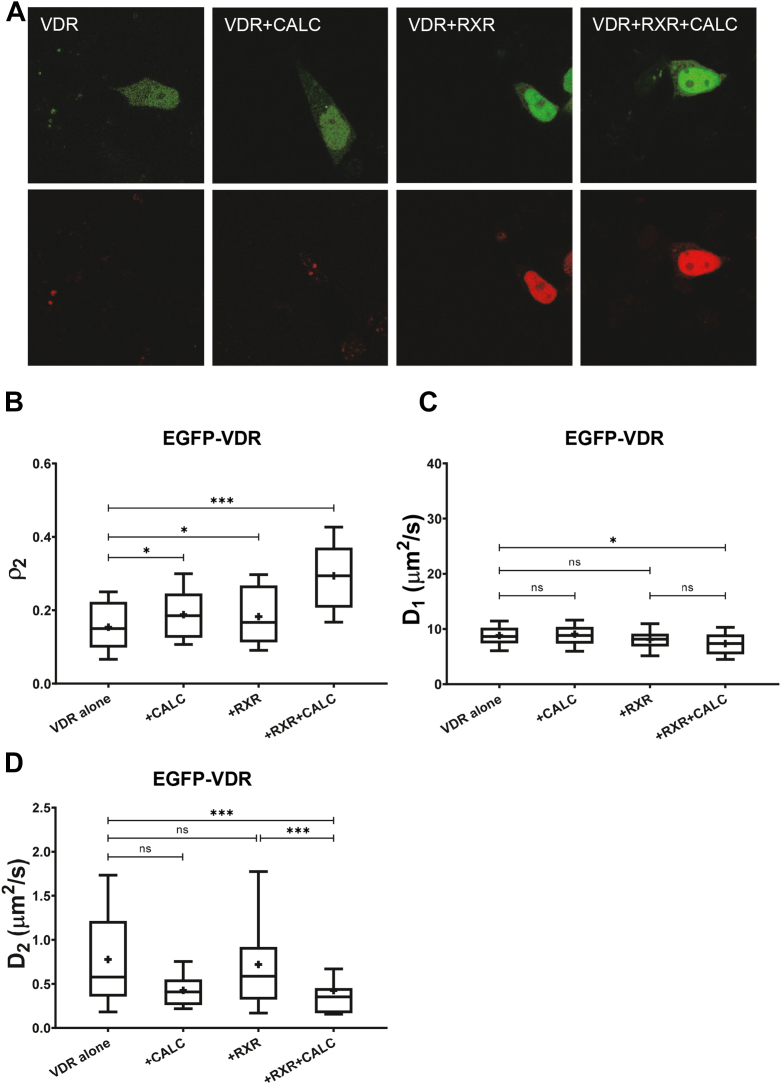
**Mobility of VDR in live HEK293 cells.***A*, representative confocal images of EGFP-VDR (*top row*) expressed alone or coexpressed with mCherry-RXR (*bottom row*) (image size: 71 × 71 μm). Where indicated, cells were treated with VDR agonist, 100 nM calcitriol (+CALC) for 20 min. *B*, fraction of the slow component of EGFP-VDR. *C*, diffusion coefficient of the EGFP-VDR fast component. *D*, diffusion coefficient of the EGFP-VDR slow component. Numbers of measurements are shown in Table S2. Boxes mark the 25th and 75th percentiles while whiskers the 10th and 90th percentile values. The *horizontal line* in the box represents the median. Averages are marked by “+.” To compare averages of ρ_2_, *t* tests were performed; the distributions of diffusion times weighted by their fractions were compared by an F-test. ∗*p* < 0.05; ∗∗∗*p* < 0.001; ns, not significant.

In contrast to EGFP-RAR, the other studied RXR partner, EGFP-VDR, had an almost homogenous distribution in the whole cell (HEK293) in the absence of ligand as shown in the confocal image in Figure 4*A*. Treatment with a saturating concentration of agonist (10^−7^ M calcitriol) or coexpression of mCherry-RXR induced nuclear accumulation (Fig. S5), similar to previous findings by us and others (21, 30, 31). The mobility of VDR was measured by FCS. Without additional cotransfection, VDR was bound to a lesser extent to DNA than RAR as suggested by the smaller slow fraction, *ρ*_*2*_ (15% *versus* 23%, see Figs. 2, *A* and *B*, 3A, and 4B), and the higher value of the diffusion coefficient *D*_*2*_ (Figs. 3*D* and 4D). Unlike in the case of RAR, agonist treatment of VDR or coexpression of RXR alone had only a slight effect on *ρ*_*2*_ and *D*_*2*_. Simultaneous addition of RXR and calcitriol enhanced DNA binding of VDR as suggested by the two-fold increase of *ρ*_*2*_ (Figs. 2*B* and 4B) and the decrease of *D*_*2*_ indicating a longer residence time on DNA (Fig. 4*D*). The mobility of the fast component decreased significantly only upon simultaneous calcitriol treatment and RXR coexpression (Fig. 4*C*). We got a similar behavior of the slow fraction of VDR in response to calcitriol treatment and RXR cotransfection in Caco-2 colorectal adenocarcinoma cells as well (Fig. S6), with significantly different endogenous transcript ratios of VDR, RAR, and RXR as compared with HEK293 (according to the Human Protein Atlas (28) (http://www.proteinatlas.org)).

### Deletion of DNA-binding domains enhances mobility of NRs

To further clarify the identity of the slow and fast components, we carried out experiments with RAR-LBD and VDR-LBD, mutants lacking their DNA-binding domains (DBDs). The slow fraction of RAR-LBD was significantly smaller than that of RAR (0.14 *versus* 0.3–0.4, see Fig. S7*A*). Thus, the slow component indeed reflects the DNA binding of RAR, which critically depends on its own DBD. The *D*_*2*_ values of the slow component were in the same range for RAR-LBD as for RAR, likely reflecting indirect DNA binding of RAR-LBD *via* an interaction partner (Fig. S7*C*). The slow fraction of RAR-LBD was not influenced by the addition of agonist or cotransfection of RXR or RXR-LBD (Fig. S7*A*). The *D*_*1*_ value of the RAR-LBD fast component was ca. 2× faster than that of RAR (7 *versus* 15 μm^2^/s) indicating that even the transient DNA binding of RAR with a short residence time depends on the DBD (Fig. S7*B*). Since the LBDs cannot bind to DNA directly, we also fitted their FCS curves with a model assuming a single diffusion component. The resulting diffusion coefficients were similar but slightly lower than those of the fast component of RAR (ca. 5 *versus* 7 μm^2^/s) and did not change significantly in response to cotransfection of RXR/RXR-LBD or treatment with AM580 RAR agonist (Fig. S7*D*). This is in line with the observed invariance of the slow fraction from the two-component fit corroborating that cotransfection of RXR or ligand treatment did not influence DNA binding of RAR-LBD.

FCS curves of VDR-LBD could be fitted well with a single diffusion component. The resulting *D* values were somewhat lower than the fast component of VDR (6.32 *versus* 8.84 μm^2^/s, see Fig. S7*E*). Contrary to the case of RAR-LBD, calcitriol treatment or RXR cotransfection led to a 40% to 50% decrease of *D* likely due to transient, indirect DNA binding of VDR-LBD *via* interaction with RXR. Cotransfection of RXR-LBD without ligand addition did not cause a decrease of *D*.

### VDR-RXR heteroassociation is enhanced by VDR agonist as indicated by FLIM-FRET

**Figure 5:**
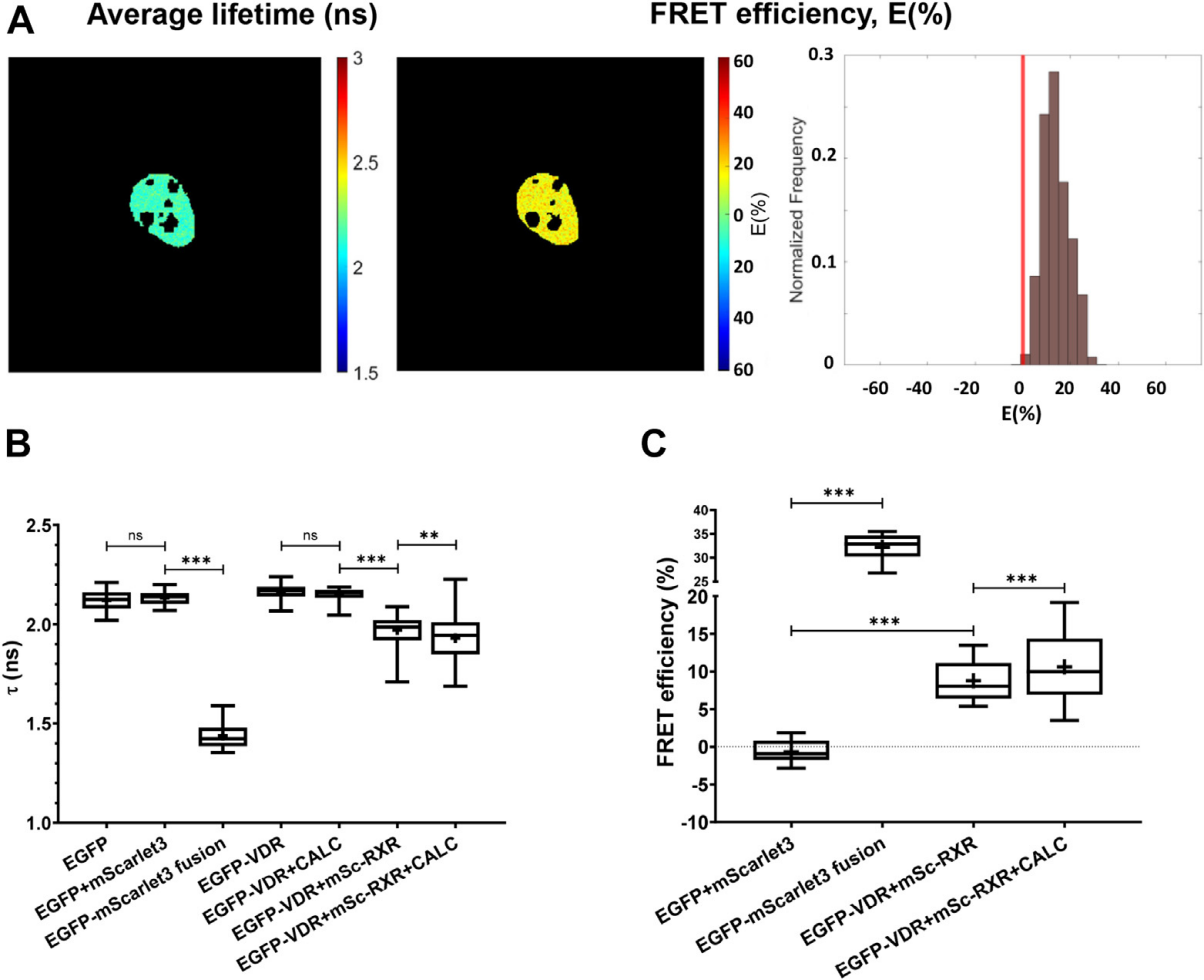
**FLIM-FRET measurements between EGFP-VDR and mScarlet3-RXR.***A*, representative donor (EGFP) fluorescence lifetime map, FRET efficiency map, and histogram of the pixelwise FRET efficiency values of the EGFP-VDR+mScarlet3-RXR sample (no ligand). Image size: 72 × 72 μm. The region of interest encircling the nucleus was selected manually. Representative lifetime and FRET images and histograms for all samples are shown in Fig. S9, lifetime decays in Fig. S10. *B*, amplitude-averaged fluorescence lifetimes of the different samples measured by FLIM. *C*, FRET efficiency values calculated from amplitude-averaged fluorescence lifetimes of the different samples. EGFP and mScarlet3 coexpressed served as a negative and the EGFP-mScarlet3 fusion protein as a positive control. Boxes mark the 25th and 75th percentiles while whiskers the 10th and 90th percentile values. The *horizontal line* in the box represents the median. Averages are marked by “+.” To compare averages, *t* tests were performed; ∗∗*p* < 0.01; ∗∗∗*p* < 0.001; ns, not significant. FLIM, fluorescence lifetime imaging microscopy; FRET, Förster resonance energy transfer.

Recently, we have reported on the molecular proximity and and comobility of RAR and RXR in response to ligand treatment using SPIM-FRET-FCCS (7). Here, we used fluorescence lifetime imaging microscopy (FLIM) to directly assess association between EGFP-VDR and mCherry- or mScarlet3-tagged RXR. If the donor (EGFP) and the acceptor (red FP) are in the 2- to 10-nm vicinity of each other, FRET occurs and shortens the fluorescence lifetime of the donor, which forms the basis of the determination of FRET efficiency; see Equation 8. mScarlet3 was introduced as an acceptor besides mCherry because it forms a better FRET pair with EGFP due to its enhanced maturation speed and efficiency and greater spectral overlap with EGFP, resulting in a larger dynamic range and higher FRET efficiencies (32). Initial measurements with EGFP and mCherry resulting in rather low FRET efficiencies are presented in Fig. S8; these indicate that calcitriol treatment increased FRET efficiency between EGFP-VDR and mCherry-RXR. FLIM measurements with mScarlet3 used as acceptor are shown in Figure 5. As an example, fluorescence lifetime and FRET efficiency maps are shown in Figure 5*A* for the EGFP-VDR+mScarlet3-RXR sample. Amplitude-averaged donor fluorescence lifetimes of the different samples are presented in Figure 5*B*. As a positive control, the fusion protein EGFP-mScarlet3 was expressed yielding *E* ∼ 32%, whereas the negative control, EGFP and mScarlet3, coexpressed as separate proteins resulted *E* = 0%. The average FRET efficiency between EGFP-VDR and mScarlet3-RXR was *E* = 9% in nontreated cells, which increased to 11% upon calcitriol treatment suggesting enhanced dimer formation (Fig. 5*C*). For the positive and negative controls, the EGFP-transfected cells, for the VDR-RXR samples, the EGFP-VDR-transfected cells (treated or not treated with calcitriol) were used as donor-only samples in FRET calculations according to Equation 9. FRET efficiency and lifetime maps for selected cells are shown in Fig. S9 for all samples, and fluorescence lifetime decays in Fig. S10.

### Agonist directed competition of VDR and RAR for RXR and DNA binding

Previously, we reported competition between NRs for heterodimerization with RXR using a nuclear translocation assay (21), which detected protein–protein interactions between NRs. The affinity of RAR to RXR was larger than that of VDR in the absence of ligand, whereas in the presence of specific agonist, always the liganded receptor dominated.

**Figure 6:**
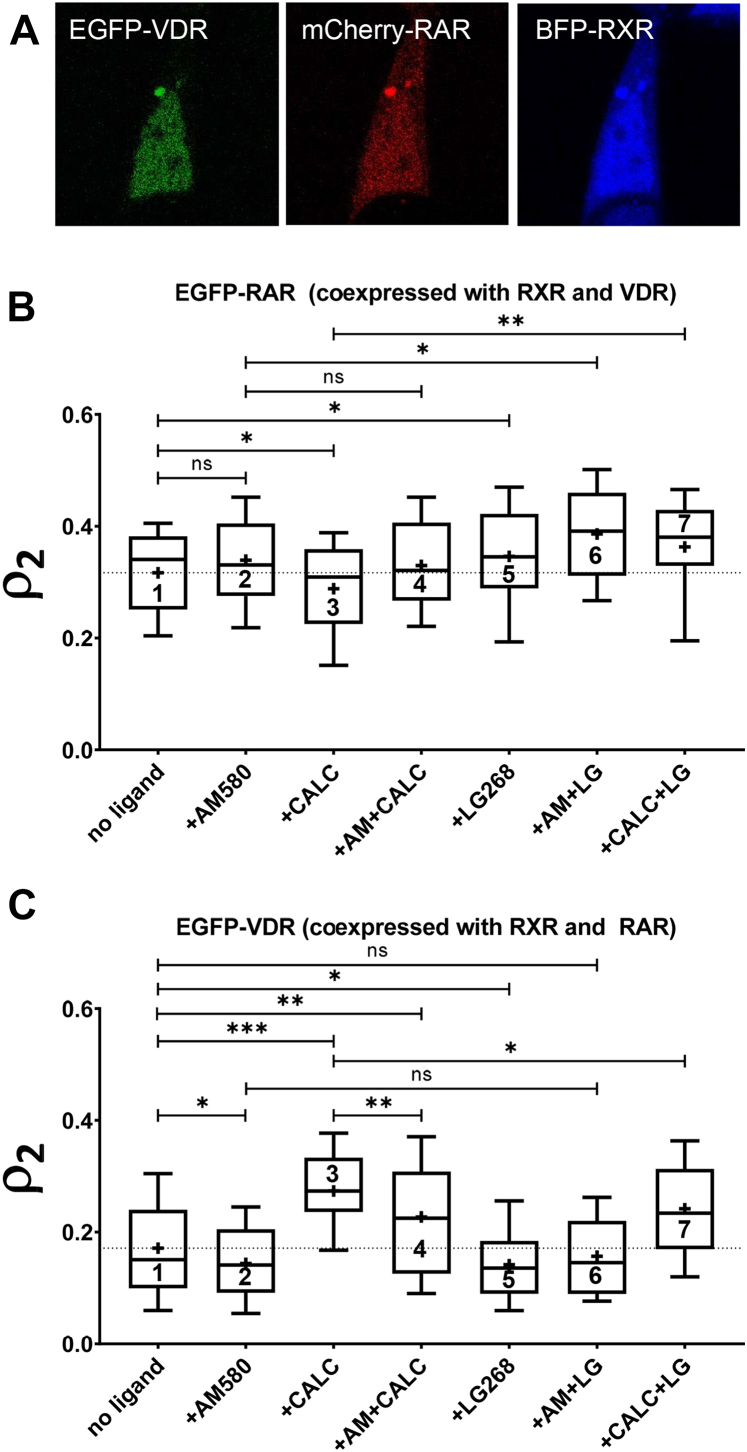
**Competition of RAR and VDR for RXR and DNA sites monitored by the fluorescence correlation spectroscopy–determined slow fraction of RAR or VDR in triple-transfected cells.***A*, confocal images of coexpressed FP-tagged receptors. EGFP-VDR and mCherry-RAR were cotransfected into HEK293^BFP-RXR^ cells stably expressing TagBFP-RXR. Image size: 41 × 41 μm. *B*, fraction of the slow component of EGFP-RAR when cotransfected with mCherry-VDR, measured in HEK293^BFP-RXR^ cells. Cells were treated with 100 nM AM580 (+AM580), 100 nm calcitriol (+CALC), 100 nM LG268 (+LG268) or with their combinations. *C*, slow fraction of EGFP-VDR when cotransfected with mCherry-RAR, measured in HEK-293^TagBFP-RXR^ cells. Column numbering is used for easier referencing of the samples in the article. Numbers of measurements are shown in Table S2. Boxes mark the 25th and 75th percentiles while whiskers the 10th and 90th percentile values. The *horizontal line* in the box represents the median. Averages are marked by “+.” To compare averages, *t* tests were performed; ∗*p* < 0.05; ∗∗*p* < 0.01; ∗∗∗*p* < 0.001; ns, not significant.

Here, we were interested whether this competition is apparent in the DNA binding of the NRs. To this end, we coexpressed all three NRs labeled with different fluorescent proteins (Fig. 6*A*). We always measured the mobility of the EGFP-tagged NR (RAR or VDR). The competing NR was labeled with mCherry and RXR with TagBFP. Cells were chosen in which the expression ratios of the three NRs were close to unity (between 0.75 and 1.25). In Figures 2, *C* and *G*, and 6B, the slow fraction of RAR is displayed in cells treated with RAR, VDR, or RXR agonists or combinations thereof. We found that, without addition of a ligand, the average RAR slow fraction was smaller in triple-transfected cells (*ρ*_*2*_ ∼ 0.32, Fig. 6*B* first column, Fig. 2*C*) than in cells transfected with RAR and RXR only (*ρ*_*2*_ ∼ 0.38, Fig. 3*A* third column, Fig. 2*A*) suggesting that a fraction of the RXR population is engaged by VDR. For VDR, which did not show increased DNA binding when coexpressed with RXR alone without ligand (Fig. 4, *B* and *E*), triple transfection with RXR and RAR did not cause a change either (see Fig. 6*C* first column and Fig. 4*B* third column).

Next, we investigated the effect of different ligands in triple-transfected cells. When applying its own agonist (AM580), we did not measure a significant change in the slow fraction of RAR (Fig. 6*B* first and second columns). It slightly decreased in the presence of VDR agonist (calcitriol), whereas in the presence of both agonists it retained the high value detected in the case of the RAR agonist alone (Fig. 6*B* third and fourth columns). The slow fraction of VDR markedly increased upon treatment with its own agonist (Fig. 6*C* first and third columns), whereas it decreased upon treatment with RAR agonist (Fig. 6*C* second column). Treatment with both agonists reduced the slow fraction of VDR to an intermediate value between those from the two independent treatments (Fig. 6*C* fourth column). This suggests that, when both ligands are present, dimerization of RXR with RAR dominates over dimerization with VDR.

The presence of an RXR agonist may also influence its heterodimerization with its NR partners and the binding of the complex to DNA. We applied its synthetic selective agonist, LG268 (10^−7^ M, saturating concentration). Based on comobility measurements, we previously found that LG268 increased RAR-RXR heterodimerization (7). Here, in triple-transfected cells, we found that LG268 enhanced the DNA-bound slow fraction of RAR and decreased that of VDR in all tested ligand combinations as described below. The DNA-bound fraction of RAR increased upon LG268 treatment as well, contrary to that of VDR, which slightly decreased (Fig. 6*B* first *versus* fifth column and Fig. 6*C* first *versus* fifth column). Thus, binding its ligand further increased the preference of RXR toward RAR *versus* VDR. When applying RXR and RAR agonists together, it further increased the DNA binding of RAR as compared with the RAR ligand alone (Fig. 6*B* second and sixth columns). On the other hand, the RXR agonist applied together with VDR agonist had an opposite effect on VDR: its DNA binding decreased as compared with VDR ligand treatment alone (Fig. 6*C* third and seventh columns). The preference of liganded RXR toward RAR is further confirmed by the effect of coadministration of RXR ligand with the ligand of the competing partner. The DNA-bound fraction of RAR increased as compared with treatment with VDR ligand alone (Fig. 6*B* third and seventh columns). For VDR, the DNA-bound fraction did not increase as compared with treatment with RAR ligand alone (Fig. 6*C* second and sixth columns).

**Figure 7:**
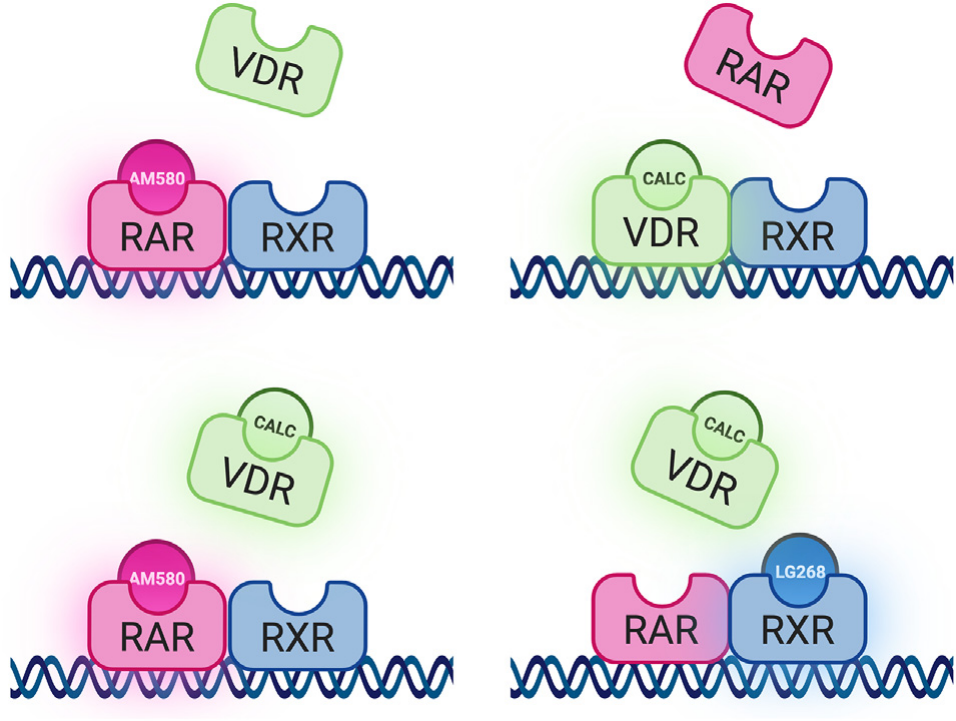
**Graphical representation of the ligand-directed competition for DNA binding between RAR and VDR.** In the presence of calcitriol (*top right*), VDR dominates DNA binding, whereas in the presence of AM580 (*top left*) or both ligands (*bottom left*), RAR does so. The presence of RXR-specific LG268 agonist (*bottom right*) favors the binding of RAR (created with BioRender.com).

Altogether, our data confirm that ligand-directed competition between RAR and VDR is present at the level of DNA binding and RAR dominates over VDR without ligand or when both RAR and VDR agonists are present. This dominance is further enhanced in the presence of the RXR ligand. Figure 7 summarizes this competition graphically.

### VDR and RAR do not heterodimerize with each other

We conducted additional experiments to exclude potential dimerization between VDR and RAR. To this end, we cotransfected cells with RAR and VDR while RXR was present only at its low endogenous concentration. The NR studied by FCS was tagged with EGFP while the other NR with mCherry.

**Figure 8:**
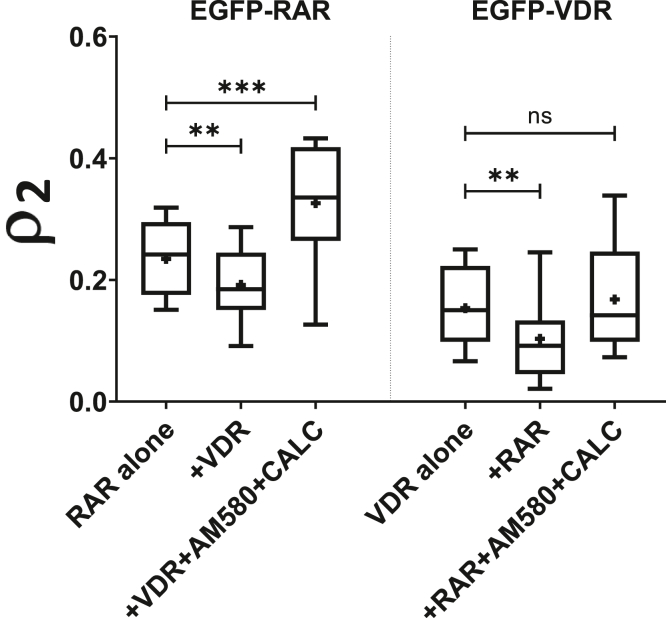
**VDR and RAR do not heterodimerize with each other.** Fraction of the slow component measured in live HEK293 cells cotransfected with RAR and VDR. The first three columns show the slow fraction of EGFP-RAR alone, coexpressed with mCherry-VDR (+VDR) or coexpressed with mCherry-VDR and treated with 100 nM AM580 and 100 nM calcitriol (+VDR+AM580+CALC). The last three columns show the slow fraction of EGFP-VDR alone, coexpressed with mCherry-RAR (+RAR), or coexpressed with mCherry-RAR and treated with 100 nM AM580 and 100 nM calcitriol (+RAR+AM580+CALC). The measured NR was always the EGFP-tagged one. Boxes mark the 25th and 75th percentiles while whiskers the 10th and 90th percentile values. The *horizontal line* in the box represents the median. Averages are marked by “+.” To compare averages, *t* tests were performed; ∗∗*p* < 0.01; ∗∗∗*p* < 0.001; ns, not significant.

In the absence of ligands, the slow fraction of the tested NR was significantly reduced when the other NR was coexpressed (Fig. 8, first *versus* second column and fourth *versus* fifth column, see also Fig. 2*E*), suggesting that these two NRs did not form a heterodimer but probably competed for endogenous RXR. Neither did ligands promote heterodimerization of these two NRs; the ligand-induced increase in DNA binding of the studied NR was not further enhanced by the presence of the other, liganded NR (Fig. 3*A* second *versus* Fig. 8 third column, and Fig. 4*B* second *versus* Fig. 8 sixth column). The dominance of RAR over VDR for dimerization with RXR was also confirmed by this set of experiments: coexpression of the other NR and double treatment with both ligands increased the slow fraction of RAR as compared with untreated RAR alone (Fig. 8 column 1 *versus* 3) but did not cause a significant change for VDR (Fig. 8 column 4 *versus* 6).

## Discussion

RXRs are promiscuous heterodimeric partners of class II NRs playing a central role in their function. The original molecular switch model of NR function assumed that NRs were bound to their response elements on the DNA statically and only the cofactors were exchanged in response to ligand binding. Biophysical experiments have shown that NRs were more dynamic and performed diffusive motion with different mobilities (6, 8, 9, 10, 12, 13, 14, 33). Previously we demonstrated that agonist binding enhanced the DNA binding of RAR and RXR in a coactivator-dependent manner (6, 14). According to our recent studies, some NR partners, RAR, VDR, and PPARγ (peroxisome proliferator-activated receptor gamma), competed for heterodimerization with RXR at the level of protein–protein interactions in HEK293 cells (21). In the absence of ligand, they displayed a hierarchy of affinities toward RXR in the order RAR>PPARγ>VDR, whereas in the presence of a specific agonist, always the liganded NR dominated. This may explain some observed side effects of systemic long-term use of NR ligand therapies such as the PPAR agonist thiazolidinediones (TZDs) against diabetes (34, 35) or retinoids applied to treat skin diseases (36). TZD treatments are associated with an increased risk of bone fractures and osteoporosis, which might be moderated by vitamin D supplementation (37). Rosiglitazone (a PPARγ-specific TZD) induces preosteoblast differentiation to adipocytes and suppresses differentiation to osteoblast *via* PPARγ signaling (38), whereas VDR enhances osteoblast function and differentiation of preosteoblast to osteoblasts *in vitro* (39); thus, the above side effect of TZDs may be at least partly due to competition between PPARγ and VDR. Rosiglitazone therapy might also interfere with retinoic acid signaling that might cause macular edema (19, 20).

To better understand the mechanism of these side effects, now we asked whether competition for RXR was also expressed at the level of DNA binding of partner NRs. Based on our results we could also refine the molecular switch model to account for ligand- and heterodimerization-induced DNA binding of NRs and the effect of competition between NRs.

With the help of modern quantitative microscopy techniques, we and others were able to decipher molecular details of the mechanism of action of transcription factors in single live cells. With such methods, the dynamic properties of interacting molecular subpopulations and proximities within molecular complexes could be dissected (6, 11, 12, 13, 14, 25, 33, 40, 41, 42). Previously, we have already confirmed the heterodimerization of RAR and RXR by demonstrating their molecular proximity and comobility using SPIM-FRET-FCCS (7). Here, we also gave direct FLIM-FRET evidence for the dimerization of VDR with RXR and its enhancement upon agonist treatment. Our FCS results presented here confirmed that RAR had a higher affinity for RXR and for DNA than VDR did. When expressed alone and in the absence of ligands, RAR had a DNA-bound slow fraction of 23% while VDR had only 15%. The slow fraction of RAR increased in the presence of RXR significantly, whereas that of VDR hardly changed. This is in line with our previous observations that, although both RAR and VDR interact with RXR in the absence of ligand (21), this interaction is stronger for RAR than for VDR. Our FCS data also implied that dimerization with RXR greatly enhanced the DNA-binding affinity of RAR but not so much that of VDR. For RAR, agonist treatment alone enhanced DNA binding; however, for VDR, simultaneous application of its agonist and coexpression of RXR were necessary to achieve this. Thus, the stronger interaction of RAR with RXR as compared with that of VDR with RXR is also manifested in the stronger DNA binding of the RAR-RXR heterodimer. We carried out similar experiments on our NRs in other cell lines: on RAR in HeLa cervical carcinoma and on VDR in Caco-2 colorectal adenocarcinoma cells. Caco-2 cells express endogenous VDR mRNA at a higher level than HEK293 cells do (7.3 *versus* 4 normalized transcript-per-million according to the Human Protein Atlas (28) (http://www.proteinatlas.org)), so it is reasonable to assume that they may possess a higher number of active VDRE response elements. We did not get qualitative differences in the behavior of the DNA-bound slow fractions in response to NR agonist treatments or RXR coexpression in the different cells indicating that our observations are not restricted to a single cell type.

The mechanism by which the presence of agonist and RXR enhanced chromatin binding was investigated by using the non-DNA-binding domains of RXR, RXR-LBD. Coexpression of RXR-LBD failed to increase the DNA-bound fraction of RAR either in the presence or in the absence of agonist. This indicates that the increase in RAR DNA binding is caused by binding of the RAR-RXR heterodimer to DNA with “two legs” and agonist contributes to this effect by promoting heterodimerization. For RAR-LBD, the DNA-non-binding variant of RAR, the slow fraction was much lower than for RAR and was not influenced by RXR contransfection or agonist treatment. When fitted with a model assuming a single diffusion component, D was practically independent of agonist and RXR cotransfection. On the other hand, when VDR-LBD was fitted with a single diffusion component, its *D* value decreased by 40% to 50% upon agonist treatment or RXR cotransfection indicating enhanced transient DNA binding with short dwell times or participation in a large complex.

Recently, we found that RXR-LBDs can form homodimers and the selective agonist LG268 increases the propensity of homodimerization (7). Chen *et al.* (43) showed by fluorescence brightness analysis that the LBDs of RAR and RXR preferentially formed heterodimers rather than homodimers upon either RAR or RXR ligand binding.

We investigated competition between NRs in cells triple transfected with RAR, VDR, and RXR *via* monitoring the DNA-bound fraction of the EGFP-tagged NR by FCS. In the absence of ligands, the RXR-induced enhancement of RAR DNA binding was slightly reduced in the presence of VDR. On the other hand, the low extent of VDR DNA binding, which was barely enhanced by RXR, was not affected by the coexpression of RAR. In our previous study, we found that the extent of dimerization of RAR with RXR was also diminished in the presence of VDR, and vice versa: the interaction between VDR and RXR decreased in the presence of RAR (21). These data suggest that competition can occur not only in protein–protein interactions but also at the level of DNA binding of NRs. Next, we studied the effect of ligands on this competition. In the presence of an RAR agonist, the DNA-bound fraction of RAR increased slightly but not significantly from the already high value, while that of VDR decreased slightly further compared with the initial very low value. VDR agonist greatly increased the DNA-bound fraction of its own receptor, while it decreased that of RAR. Simultaneous application of both agonists restored the high DNA-bound fraction of RAR measured in the presence of RAR agonist alone. For VDR, the DNA-bound fraction increased as compared with the case of RAR agonist treatment alone but did not reach the value attained with calcitriol treatment alone. RXR agonist (LG268) further enhanced the increase in RAR's AM580-induced DNA binding and partially compensated for the calcitriol-induced decrease, whereas in the case of VDR, it reduced the calcitriol-induced increase in DNA binding. This suggests that the RXR ligand enhances heterodimerization of RXR with RAR over VDR. These results are consistent with our previous observations on protein–protein interactions between NRs and their dependence on ligand treatments (21). In the absence of ligands or in the presence of both NR ligands, heterodimerization with RXR and DNA binding are dominated by RAR over VDR, whereas when a single ligand is added, the liganded receptor dominates these interactions.

Different cell types express RXR and other partner NRs in different relative amounts. In cell types where RXR is abundant and thus not limiting: (RAR+VDR)<<RXR, no competition for RXR can be expected. The strength/affinity of pairwise interactions between NRs is probably not influenced by cell type; however, the relative expression level of NRs, the availability of NR-specific coactivators, and the number of NR-specific response elements might influence the DNA binding of NRs and the outcome of competition in different cell types.

In our experiments we chose cells with approximately equal expression levels of the different FP-tagged NRs, which is optimal for detecting competition. In experiments where interactions of fluorescently tagged proteins are studied with a background of endogenously expressed proteins, the transfected to endogenous expression ratios must be taken into account. In HEK293 cells used for FCS, the transfection of EGFP-RAR and EGFP-VDR increased the total available RAR and VDR pool by 21% and 27%, respectively; thus, overexpression did not significantly perturb the physiological status. Transfection of mCherry-RXR or BFP-RXR equaling the amount of the other two FP-NRs increased the RXR pool by ∼120% suggesting that in nontransfected samples the amount of RXR is indeed limiting and explains the significant increase of the DNA-bound fractions of RAR and VDR.

We have previously shown that the action of an NR was hindered by the application of a competing NR’s ligand as seen from gene expression changes and cellular responses. Treatment of undifferentiated mouse embryonic stem cells with retinoic acid reduced the expression of liver X receptor (LXR) target genes and the binding of RXR to LXR response elements while inducing the recruitment of RXR to RAR target genes. This ligand-induced switch in RXR preference from LXR to RAR is critical for proper cell differentiation (44) indicating that ligand-directed competition between NRs manifested in gene expression may influence cell fate.

Previously, we proposed a model for the formation of activating NR complex (7, 21), in which all elements of the complex contribute to its stability. Recently, a cooperative binding model for the formation of the ternary complex of PPARγ, its ligand, and its coregulator was published (45). Our new results add yet another aspect to the molecular switch model: NRs compete not only for RXR but also for binding sites on the DNA, in a ligand-dependent manner. DNA binding of RAR exceeds that of VDR in the absence of ligand or in the presence of agonists of both NRs, while in the presence of one agonist the liganded NR dominates. When RAR and VDR are both present, RXR agonist promotes DNA binding of the RAR-RXR rather than the VDR-RXR heterodimer. Altogether, NR dimerization, agonist binding, and coactivator binding collectively enhance the DNA binding affinity of NRs, which may compete for RXR and binding sites. The processes of dimerization, coactivator binding, and DNA binding enhance each other, and together with ligand binding they jointly contribute to the stability of the regulatory complex.

We demonstrated that the ligand-directed competition of NRs for RXR was also manifested in their DNA binding, supporting our notion that this competition may be responsible for side effects of long-term systemic application of NR agonists. This could raise alternative medical strategies; applying cotreatments could alleviate side effects due to overactivating a single NR pathway at a time (46). This calls the attention to the necessity of combined and time-shifted administration of NR ligands in therapies.

## Experimental procedures

### Plasmid design

cDNAs coding for full-length RARα, RXRα (referred to as RAR and RXR in this article) were purchased from the cDNA Resource Center, subcloned after PCR amplification into modified pEGFP-C3 (Clontech Laboratories) and pmCherry-C3 vectors using XhoI and HindIII sites. RAR and RXR ligand-binding domain sequences were acquired from UniProt (47). NR LBD (ligand-binding domain, lacking DBD) sequences were amplified using PCR, then subcloned into pEGFP-C3 (Clontech Laboratories) and pmCherry-C3 vectors using the same technique as that used for the full-length receptors. The EGFP-VDR construct was made in our department as described in a previous paper (21).

pLenti-C-Myc-DDK-IRES-Neo, a third-generation transfer vector (Origene) was digested with EcoRI and PmeI and subcloned with HaloTag, EGFP, and RARα sequence. Then it was modified by cutting out the HaloTag to suit cloning the NRs and the fluorescent proteins used in our laboratory. To clone mScarlet3-RXR, the newly designed pLenti-C-Myc-DDK-EGFP-RARα transfer vector was used. By using BsTB1 and Mlu1, EGFP was replaced by mScarlet3, then RXRα was inserted between MluI and Pmel using proper primers.

### Cell culture and transient transfection

HEK293 (human embryonic kidney), HeLa (human cervix carcinoma), and Caco-2 (human colorectal adenocarcinoma) cells were maintained in phenol red–containing Dulbecco’s modified Eagle’s medium supplemented with 10% fetal bovine serum (Sigma-Aldrich), GlutaMAX (Fisher Scientific), and 50 mg/l gentamycin (KARA). Cells were passaged every 3 days and held in a humidified atmosphere at 37 °C and 5% CO_2_. Forty-eight hours before measurements, cells were plated on 8-well ibidi plates with coverslip bottom (ibidi) at a concentration of 15,000 cells/well in 300 μl phenol red–free Dulbecco’s modified Eagle’s medium. Twenty-four hours prior to measurements, cells were transfected with 65 ng of plasmids coding for NRs N-terminally tagged with EGFP or mCherry using 0.3 ng FuGene HD transfection reagent (Promega) following the user’s guide of the manufacturer. For competition measurements, a HEK293 cell line stably expressing TagBFP-RXRα was used established earlier in our laboratory (21). Transfections by fluorescently marked RARα and VDR receptor were carried out as described above. Measurements took place 24 h after transfection.

Relative expression levels of FP-tagged NRs were determined from confocal images by comparing fluorescence intensities with those of EGFP-mCherry and TagBFP-EGFP (or EGFP-TagBFP) fusion proteins expressing the two dyes at a 1:1 ratio. The preparation of the plasmids of these fusion proteins is described elsewhere (7). Molecular expression ratios of cotransfected NRs were calculated as described in our previous papers (21, 25, 26). Representative images of HEK293 cells coexpressing EGFP-RAR or EGFP-VDR with mCherry-RXR are shown in Fig. S11.

### Western blot

Transfected and control cells were lysed in RIPA lysis buffer (Santa Cruz Biotechnology Inc) supplemented with 1× protease inhibitor cocktail and 1 mM PMSF according to the manufacturer's instructions, and then total cellular protein of 10^5^ cells/well was separated on 10% SDS–polyacrylamide gel and electroblotted onto a 0.45-μm-pore-size PVDF membrane. The blot was saturated with milk blocking buffer (5% milk powder/0.2% Tween-20/PBS) for 1 h, and then VDR, RARα, RXRα, and β-actin were labeled by mouse polyclonal anti-VDRα (sc-13133), anti-RARα (sc-515796), anti-RXRα (sc-515929), and anti-actin (sc-8432) primary antibodies (all from Santa Cruz) applied at a final concentration of 1 μg/ml in milk blocking buffer overnight at 4 °C. After washing with 0.2% Tween-20/PBS for 6 × 5 min, the membrane was incubated with goat anti-mouse IgG secondary antibodies conjugated with horseradish peroxidase (ab6789 from Abcam) at a final concentration of 2 μg/ml in milk blocking buffer for 1.5 h at room temperature. After washing with 0.2% Tween-20/PBS for 6 × 5 min, the bands were visualized with SuperSignal West Pico PLUS Chemiluminescent Substrate (cat. # 34577, Thermo Fisher Scientific) and detected by a luminescence reader (FluorChem Q System, Alphaview Gel Documentation System, Alpha Innotech Corporation). The protein content of immunoreactive bands was quantified with densitometry as described in the next section.

### Determination of the relative expression levels of endogenous and transfected NRs in FCS samples

The relative expression ratio of FP-tagged (transfected) and nontagged (endogenous) NRs in cells used for FCS measurements was determined by a combination of Western blot and confocal microscopy analysis. First, we used Western blot as described above (see Fig. S1, *A*–*C*) to determine the expression ratios of FP-tagged to nontagged NRs in the total cell population. Integrated intensities of immunoreactive bands corresponding to the NR species in bioluminescence images were measured by using Fiji (48) and were corrected for background measured at areas outside the bands.

For FCS, cells having low fluorescence intensities were used. The FP-NR expression level of these cells relative to the average of the total cell population was calculated from fluorescence intensities in confocal overview images recorded with a zoom factor of 1 (image size: 212 × 212 μm, ca. 150 cells/sample). From these parameters (see Fig. S1*D*), the expression ratios of FP-tagged to endogenous nontagged NRs (in cells used for FCS measurements) were calculated as:

(1) $$\frac{c\left({NR}_{FP}\right)}{c\left({NR}_{endog.}\right)}=\frac{I_{{NR}_{FP}}^{WB}}{I_{{NR}_{endog.}}^{WB}}\times \frac{\left\langle F_{FCS}\right\rangle }{\left\langle F_{total}\right\rangle }$$

where *c(NR*_*FP*_*)*/*c(NR*_*endog.*_*)* is the average concentration ratio of the FP-tagged and endogenous NRs in cells typically selected for FCS measurements, $I_{{NR}_{FP}}^{WB}$ and $I_{{NR}_{endog.}}^{WB}$ are the background-corrected bioluminescence intensities of the bands corresponding to FP-tagged and endogenous NRs from Western blots, and $\left\langle F_{FCS}\right\rangle$ and $\left\langle F_{total}\right\rangle$ are the average fluorescence intensities per pixel for cells typically selected for FCS analysis and for the total cell population (including transfected and nontransfected cells) in the microscopy sample. Western blot and confocal microscopic measurements were carried out on the same transfected cell populations simultaneously to ensure comparability.

The thus determined transfected to endogenous NR ratios in cells used for FCS experiments were 0.21 for EGFP-RAR/RAR, 0.27 for EGFP-VDR/VDR, and 1.19 for mCherry-RXR/RXR.

### Ligand treatment

We used AM580 and LG268 synthetic agonist ligands (BioVision Inc and Sigma Aldrich) for the activation of RAR and RXR, respectively, and calcitriol (BioVision Inc) for VDR. Original stocks were diluted in dimethyl sulfoxide. We created working solutions in 50-50% ethanol-dimethyl sulfoxide mixture and stored them at −20 °C in small amounts sufficient for one measurement to prevent repeated freezing and thawing of the ligand. Ligands were added to the cells at a final concentration of 100 nM and incubated at 37 °C for 20 min. Measurements were then carried out within 1 h, after which a new set of cells was used.

### Confocal fluorescence correlation spectroscopy

In FCS, fluorescence intensity fluctuations, caused by the diffusion of fluorescently tagged molecules in a sub-femtoliter-sized volume are detected. From the fluctuating fluorescence signal, an ACF is calculated, which reflects the photophysical and diffusion properties of molecules (23). By fitting the autocorrelation curves with different models, the diffusion coefficients and the fractions of molecular subpopulations can be obtained (22, 24, 49). First, confocal images were taken from the cells on a Zeiss LSM880 laser-scanning confocal microscope (Carl Zeiss) using 40× water immersion objective, NA 1.2. BFP was excited by a 405-nm diode laser, and the blue fluorescence signal was detected between 415 and 490 nm. EGFP was excited by the 488-nm line of an Argon-Ion laser and detected between 500 and 534 nm. mCherry was excited by a HeNe laser at 543 nm and detected between 578 and 696 nm. FCS measurements were carried out with the EGFP-tagged NRs using the 488-nm line. The laser intensity at the microscope objective was measured before each measurement and set to 1.7 μW. In each cell, ten runs with 8-s duration were recorded at two selected spots in the nucleus. Measurements were carried out at room temperature (22.5 °C).

### Evaluation of raw FCS data

FCS data were evaluated by using the QuickFit3 software (https://biii.eu/quickfit-3). ACFs from each run were inspected, and those displaying artefacts due to, *e.g.*, cell movements, or large fluctuations caused by aggregates were excluded. The remaining runs were averaged, and the resulting correlation curve was fitted to different models using a simulated annealing algorithm with box constraints weighted by the standard deviations of the runs. We tested normal (free Brownian) and anomalous diffusion models with one or two components to fit ACFs. Each model included triplet and EGFP blinking terms:

(2) G(τ)=1−T−Φc+Te−ττtr+Φce−ττc1−T−ΦcGdiff(τ)

where

(3) $$G_{diff}^{normal}\left(\tau \right)=\frac{1}{N}\left[\rho_{1}{\left(1+\frac{\tau }{\tau_{1}}\right)}^{-1}{\left(1+\frac{\tau }{{S^{2}\tau }_{1}}\right)}^{-1/2}+\rho_{2}{\left(1+\frac{\tau }{\tau_{2}}\right)}^{-1}{\left(1+\frac{\tau }{{S^{2}\tau }_{2}}\right)}^{-1/2}\right]$$

and

(4) $$G_{diff}^{anom}\left(\tau \right)=\frac{1}{N}\left[\rho_{1}{\left(1+{\left(\frac{\tau }{\tau_{1}}\right)}^{\alpha_{1}}\right)}^{-1}{\left(1+{\left(\frac{\tau }{{S^{2}\tau }_{1}}\right)}^{\alpha_{1}}\right)}^{-1/2}+\rho_{2}{\left(1+{\left(\frac{\tau }{\tau_{2}}\right)}^{\alpha_{2}}\right)}^{-1}{\left(1+{\left(\frac{\tau }{{S^{2}\tau }_{2}}\right)}^{\alpha_{2}}\right)}^{-1/2}\right]$$

*N* denotes the average number of diffusing fluorescent molecules present in the detection volume, *τ* is the lag time, *T* is the equilibrium mole fraction of fluorophores in triplet state, and *τ*_*tr*_ is the triplet correlation time (fixed to 3 μs) (50). For EGFP two independent protonation mechanisms—intramolecular proton transfer and pH-dependent external protonation—were described (51). Since the characteristic time constants of the two protonation processes are separated by less than an order of magnitude at pH 7.4, a single term, characterized by the molecular fraction *Φ*_*c*_ and the correlation time *τ*_*c*_ (fixed to 320 μs) was considered (52).

In the normal and anomalous diffusion models, we assumed one or two distinct species: a fast population with a fraction of *ρ*_*1*_ and a diffusion time of *τ*_*1*_ and a slow one with a fraction of *ρ*_*2*_ and a diffusion time of *τ*_*2*_; *ρ*_*2*_ equals 1 − *ρ*_*1*_. *α*_1_ and *α*_2_ are the anomaly parameters of the two species; for normal diffusion they equal 1, for anomalous subdiffusion, which can be caused, *e.g.*, by molecular crowding or the presence of large obstacles, they are <1. For fitting ACFs of NRs, we selected the two-component, whereas for EGFP monomers and dimers, the one-component normal diffusion model; goodness of fit (χ^2^) values for NRs are listed in Table S1. *S* is the ratio of the axial and longitudinal diameters of the ellipsoid-shaped confocal detection volume, defined by the properties of the microscope. *S* was determined before each measurement by fitting the ACFs of the Alexa Fluor 488 dye solution.

The diffusion coefficients of the fast and slow components were calculated as:

(5) $$D_{i}={\omega_{xy}}^{2}/4\tau_{i}$$

where *ω*_*xy*_ is the lateral radius of the detection volume. *ω*_*xy*_ was calculated from the measured diffusion time of 10 nM Alexa Fluor 488 dye in 10 mM Tris-EDTA buffer, pH 7.4 as follows:

(6) $$\omega_{xy}=\sqrt{4D\tau_{D}}$$

where *τ*_*D*_ is the diffusion time of the dye and *D* is its diffusion coefficient taken from the literature (414 μm^2^/s at 25 °C) (53).

The number of cells measured for each condition is listed in Table S2.

### FLIM-FRET measurement

Heterodimerization between EGFP-VDR and mScarlet3-RXR was monitored by Förster resonance energy transfer (FRET). FRET is the nonradiative transfer of energy from an excited donor dye (here, EGFP) to a nearby acceptor (mScarlet3) *via* dipole–dipole coupling. The FRET efficiency, *E*, which is the probability that an excited donor transfers its energy to an acceptor, decreases with the negative sixth power of their distance and has a range of 2 to 10 nm making FRET an ideal tool for indicating molecular associations. The fluorescence lifetime of the donor is shortened in the presence of an acceptor, which is the basis for the determination of *E* by FLIM (54). For FLIM measurements, a time-correlated single photon counting upgrade kit (PicoQuant) coupled to an A1 confocal microscope (Nikon) equipped with a Plan-Apochromat 60×/NA 1.27 water immersion objective was used. EGFP fluorescence was excited by a 485-nm picosecond pulsed laser with a repetition rate of 20 MHz. Emission was detected through a 520/35-nm emission filter using a PMA hybrid 40 photon-counting photomultiplier. Data were collected for 90 s. For fluorescence lifetime analysis, images were first intensity thresholded to exclude cell-free areas, then the nuclei were selected with a free-hand-drawn region of interest excluding nucleoli. Fluorescence intensity decays integrated for a whole region of interest were analyzed; for selected cells, analysis was also carried out on a pixel-by-pixel basis and presented as lifetime maps. The resulting fluorescence decay curves were fitted with the SymphoTime 64 software to a multiexponential reconvolution model with two lifetime components:

(7) $$y\left(t\right)=\sum_{i=1}^{2}IRF\;⨂{|}_{{Bkgr}_{IRF}|{Shift}_{IRF}}\;A\left[i\right]\;\exp\left(-\frac{t}{\tau \left[i\right]}\right)+{Bkgr}_{Dec}$$

(8) $$\tau_{AvAmp}=\frac{\sum_{i=1}^{2}A\left[i\right]\tau \left[i\right]}{\sum_{i=1}^{2}A\left[i\right]}$$

where *A* [*i*] is the amplitude, τ [*i*] is the exponential decay time of the *i*^th^ component, *Bkgr*_*Dec*_ is correction for decay background, Bkgr_IRF_ is the correction for background of the instrument response function (IRF), Shift_IRF_ is the correction for temporal IRF displacement, and *τ*_*AvAmp*_ is the amplitude-weighted average lifetime. The FRET efficiency was determined by the following equation:

(9) $$E=1-\frac{\tau_{DA}}{\tau_{D}}$$

where *τ*_*DA*_ is the amplitude-weighted average lifetime of the donor in the donor-acceptor-tagged samples and *τ*_*D*_ is that of the donor-only sample (https://www.picoquant.com/images/uploads/page/files/7267/appnote_flim_fret.pdf). For FRET measurements between EGFP-VDR and mScarlet3-RXR, EGFP-VDR expressed alone was used as a donor-only sample. As a positive control for FRET, we used the EGFP-mScarlet3 fusion protein with a 6-amino acid linker between the two dyes, while as a negative control, EGFP and mScarlet3 were coexpressed as separate proteins. For these control samples, EGFP alone was expressed as a donor-only sample.

For FLIM measurements, we first used mCherry as an acceptor instead of mScarlet3, which resulted in low FRET efficiencies between EGFP-VDR and mCherry-RXR. Therefore, we switched to mScarlet3, which is blue-shifted relative to mCherry, resulting in a larger spectral overlap between donor emission and acceptor absorption spectra, and also its maturation is enhanced as compared with mCherry; both factors lead to a larger Förster radius (donor–acceptor distance at which E = 0.5) and higher FRET efficiencies. We estimated the Förster radius for EGFP-mCherry to be 5.37 nm and for EGFP-mScarlet3 5.76 nm (assuming κ^2^ = 2/3 for the orientation factor and n = 1.333 for the refractive index of the medium). We estimate that the maturation extent for mScarlet3 is 94.9%/70.9% = 1.33 times better than for mCherry (HeLa cells 24 after transfection) (32).The FLIM-FRET data obtained with the EGFP-mCherry dye pair are given in Fig. S8.

### Statistical evaluation

The statistical analysis of our data was performed using GraphPad Prism (version 8.0.0 for Windows, GraphPad Software, www.graphpad.com). To compare the goodness of fits for the four tested fitting models (one-component normal, one-component anomalous, two-component normal, two-component anomalous diffusion), average *χ*^*2*^ values were obtained, then we applied Akaike information criterion (55) and F-test (two-component normal *versus* one-component anomalous diffusion) using GraphPad QuickCalcs website. (https://www.graphpad.com/quickcalcs/aic1/). The best fit for full-length NRs was given by the two-component anomalous diffusion model followed by the two-component normal diffusion model. We used the latter model for fitting ACFs of full-length NRs because it grasped the main features of NR behavior, while the anomalous model increased the scatter of the fit parameter values to a great extent.

The distributions of diffusion times or diffusion coefficients from two-component fits weighted by their fractions were compared by an F-test, using a custom-written C++ software. Comparison of the averages of *ρ*_*2*_ values was carried out using unpaired *t* tests.

## Data availability

All microscopy data are available upon request from Dr György Vámosi (vamosig@med.unideb.hu).

## Supporting information

This article contains supporting information.

## Conflict of interest

The authors declare that they have no conflicts of interest with the contents of this article.
