# Supplementary material for: Agonist-controlled competition of RAR and VDR nuclear receptors for heterodimerization with RXR is manifested in their DNA binding
Source: J Biol Chem. 2023 Jan 11;299(2):102896. doi: 10.1016/j.jbc.2023.102896 (PMC9943875; doi:10.1016/j.jbc.2023.102896)
Supplement: Supplemental Figures S1–S11 and Table S1 [file mmc1.pdf]

Supporting Information

Agonist-controlled competition of RAR and VDR nuclear receptors for heterodimerization with RXR is manifested in their DNA-binding

Bálint Rehó[1], Lina Fadel[1], Peter Brazda[2,3], Anass Benziane[1], Éva Hegedüs[1], Pialy Sen[1], Theodorus W. J. Gadella[4], Katalin Tóth[1], László Nagy[2,5,6], György Vámosi[1,6]

[1]Department of Biophysics and Cell Biology, Doctoral School of Molecular Medicine, Faculty of Medicine, University of Debrecen, Debrecen, Hungary

[2]Department of Biochemistry and Molecular Biology, Faculty of Medicine, University of Debrecen, Debrecen, Hungary

[3]Princess Maxima Centre for Pediatric Oncology, Utrecht, The Netherlands

[4]Section of Molecular Cytology and van Leeuwenhoek Centre for Advanced Microscopy (LCAM), Swammerdam Institute for Life Sciences, University of Amsterdam, Amsterdam, the Netherlands

[5]Johns Hopkins University School of Medicine, Department of Medicine and Biological Chemistry, Institute for Fundamental Biomedical Research, Johns Hopkins All Children's Hospital, Saint Petersburg, FL 33701, USA

χ^2 values of different fits

Sample name	Ligand	One component, normal	One component, anomalous	Two component, normal	Two component, anomalous
RAR		49.92	16.84	15.18	13.03
RAR	+AM580	21.65	17.50	13.90	12.22
RAR+RXR		67.98	18.15	17.57	17.76
RAR+RXR	+AM580	47.53	20.33	12.65	11.69
RAR+RXR-LBD		59.24	16.75	14.63	13.61
RAR+RXR-LBD	+AM580	61.65	16.92	13.78	12.01
VDR		41.58	14.29	14.88	12.78
VDR	+Calcitriol	44.17	15.42	14.09	11.91
VDR+RXR		43.98	13.75	13.21	10.71
VDR+RXR	+Calcitriol	47.71	18.50	15.02	12.71
RAR+RXR+VDR		62.07	20.48	14.38	11.87
RAR+RXR+VDR	+AM580	68.63	19.85	14.60	13.32
RAR+RXR+VDR	+Calcitriol	51.83	16.89	14.05	12.20
RAR+RXR+VDR	+LG268	51.51	24.94	14.92	12.77
RAR+RXR+VDR	+AM580<br>+LG268	41.84	19.93	16.15	14.14
RAR+RXR+VDR	+LG268<br>+Calcitriol	53.64	21.63	13.19	13.15
RAR+RXR+VDR	+AM580<br>+Calcitriol	58.58	20.72	14.92	13.06
VDR+RXR+RAR		49.76	17.87	14.10	12.37
VDR+RXR+RAR	+AM580	42.18	16.00	13.75	12.21
VDR+RXR+RAR	+Calcitriol	62.88	19.65	15.64	13.30
VDR+RXR+RAR	+LG268	45.59	16.24	15.61	13.53
VDR+RXR+RAR	+AM580<br>+LG268	43.22	16.78	13.54	12.51
VDR+RXR+RAR	+LG268<br>+Calcitriol	54.97	18.38	17.06	14.93
VDR+RXR+RAR	+AM580<br>+Calcitriol	52.10	20.61	15.94	13.24

Table S1. Table showing the calculated χ^2 values (goodness of fit) of samples with 1-component normal, 1-component anomalous, 2-component normal, and 2-component anomalous diffusion models with triplet state formation and blinking of EGFP. Fits were compared using Akaike's information criterion. The best fit was given by the two-component anomalous diffusion model followed by the 2-component normal diffusion model. We used the latter model for fitting ACFs of full length NRs because it grasped the main features of NR behavior, while the anomalous model increased the scatter of the fit parameter values to a great extent.

Number of measurements

Sample name	Ligand	Number of measured cells
RAR		40
RAR	+AM580	37
RAR+RXR		30
RAR+RXR	+AM580	25
RAR+RXR-LBD		25
RAR+RXR-LBD	+AM580	26
VDR		49
VDR	+Calcitriol	39
VDR+RXR		46
VDR+RXR	+Calcitriol	40
RAR+RXR+VDR		74
RAR+RXR+VDR	+AM580	55
RAR+RXR+VDR	+Calcitriol	68
RAR+RXR+VDR	+LG268	69
RAR+RXR+VDR	+AM580 +LG268	32
RAR+RXR+VDR	+LG268 +Calcitriol	29
RAR+RXR+VDR	+AM580 +Calcitriol	25
VDR+RXR+RAR		91
VDR+RXR+RAR	+AM580	84
VDR+RXR+RAR	+Calcitriol	79
VDR+RXR+RAR	+LG268	91
VDR+RXR+RAR	+AM580 +LG268	46
VDR+RXR+RAR	+LG268 +Calcitriol	38
VDR+RXR+RAR	+AM580 +Calcitriol	52

Table S2. Number of cells measured in each sample.

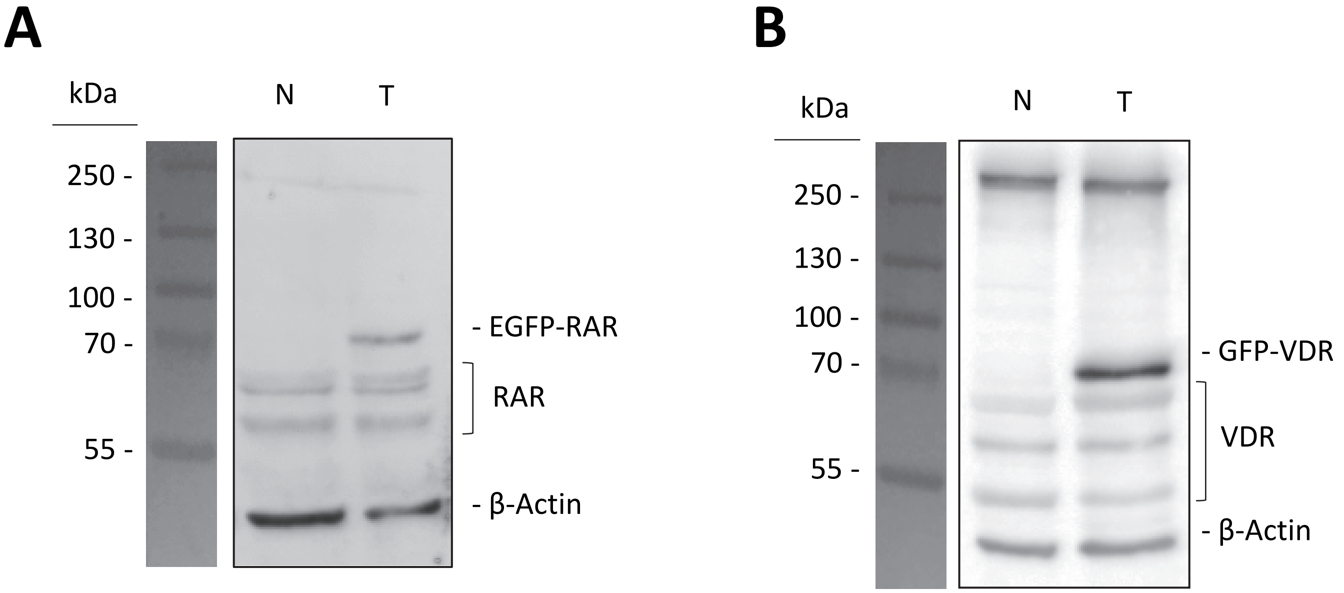

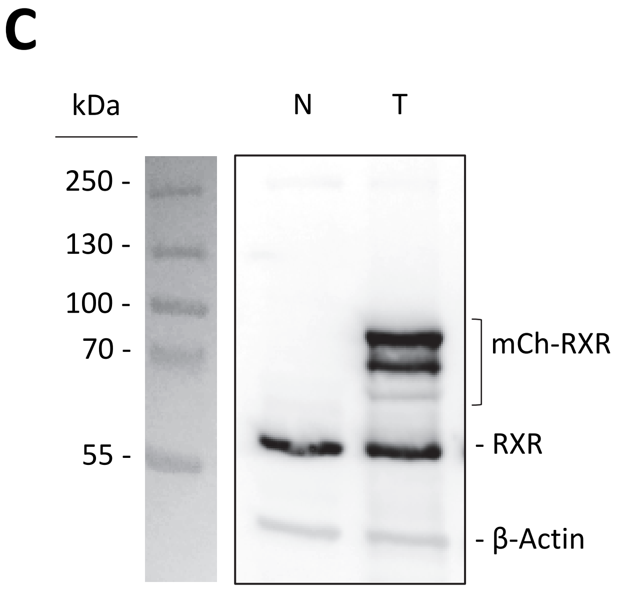

D

	$I^{WB}_{NR_{FP}}/I^{WB}_{NR_{endog.}}$	$\langle F_{FCS}\rangle/\langle F_{total}\rangle$	$\mathbf{c(NR_{FP})/c(NR_{endog.})}$
RAR	0.67	0.32	**0.21**
VDR	1.49	0.18	**0.27**
RXR	2.12	0.56	**1.19**

Fig. S1. Western-blot analysis of non-tagged endogenous and FP-tagged transfected NRs expressed in HEK293 cells. Left lanes: non-transfected cells (N) expressing only endogenous NR; right lanes: cells transfected with FP-NR (T). β-actin was used as a control. A) Non-transfected and EGFP-RAR-transfected sample. B) Non-transfected and EGFP-VDR-transfected sample. C) Non-transfected and mCherry-RXR-transfected sample. Expected masses, RAR - 51, VDR - 48, RXR - 50-54, EGFP - 27, mCherry - 28, β-actin - 42 kDa. Integrated densitometric values of the indicated bands were used for assessing relative expression ratios of FP-tagged to endogenous non-tagged NRs in the whole cells population. D) Expression ratios of FP-tagged to endogenous non-tagged NRs (in cells used for FCS measurements), where $c(NR_{FP})/c(NR_{endog.})$ is the average concentration ratio of the FP-tagged to endogenous NRs in cells typically selected for FCS measurements, $I^{WB}_{NR_{FP}}/I^{WB}_{NR_{endog.}}$ the ratio of background-corrected bioluminescence intensity of the bands corresponding to FP-tagged and endogenous NRs from Western-blots, and $\langle F_{FCS}\rangle/\langle F_{total}\rangle$ is ratio of the average fluorescence intensity per pixel for cells typically selected for FCS analysis to that for the total cell population (including transfected and non-transfected cells) in the microscopy sample.

EGFP-RAR fits with 4 different models

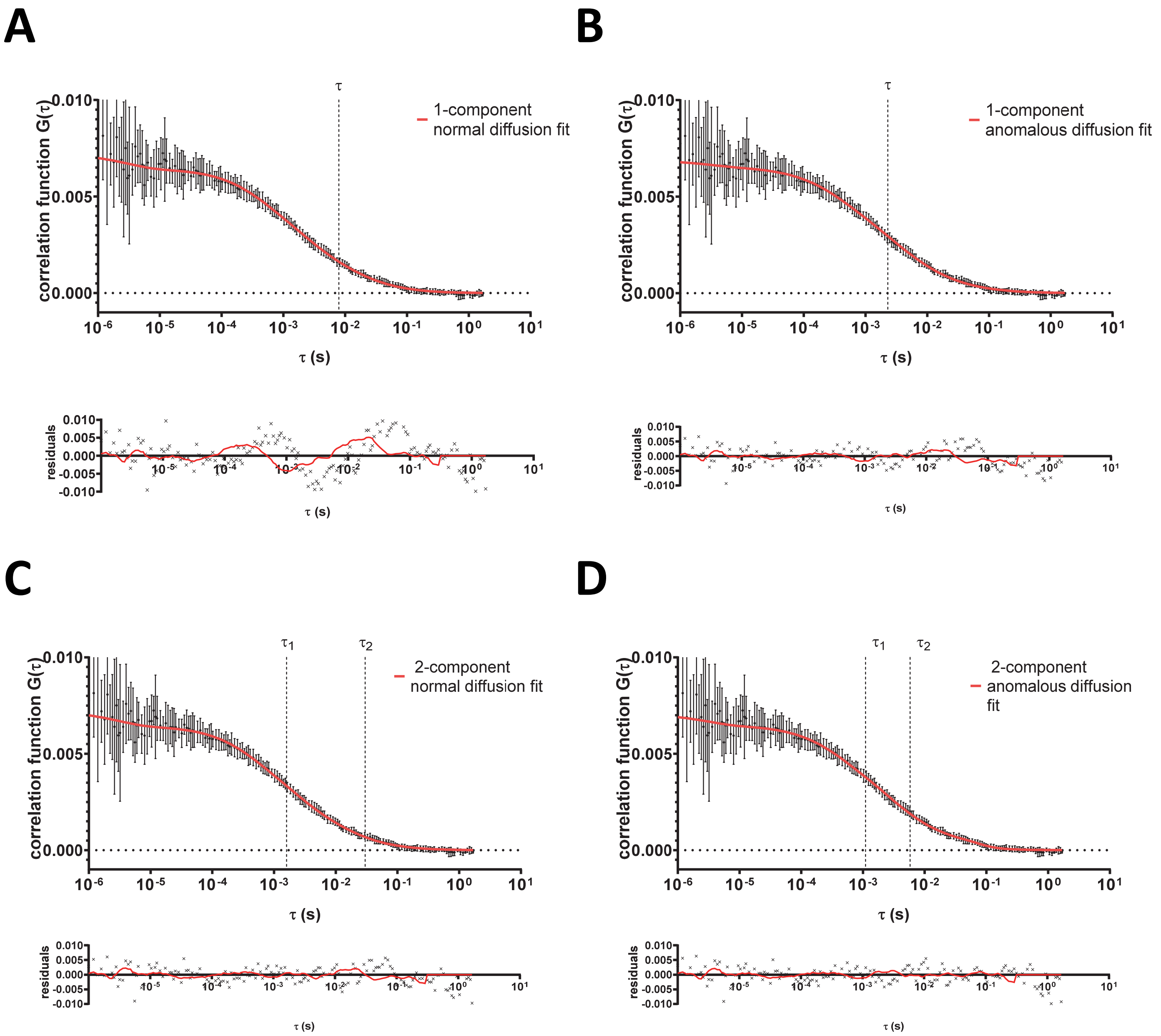

Fig. S2. Representative fits of EGFP-RAR with different model functions. A) 1-component normal diffusion, B) 1-component anomalous diffusion, C) 2-component normal diffusion – this model was selected for full length NRs, displayed also in Fig. 1B, D) 2-component anomalous diffusion model. All models include triplet state formation and blinking of EGFP. τ-s indicate the diffusion time(s) obtained from the fits.

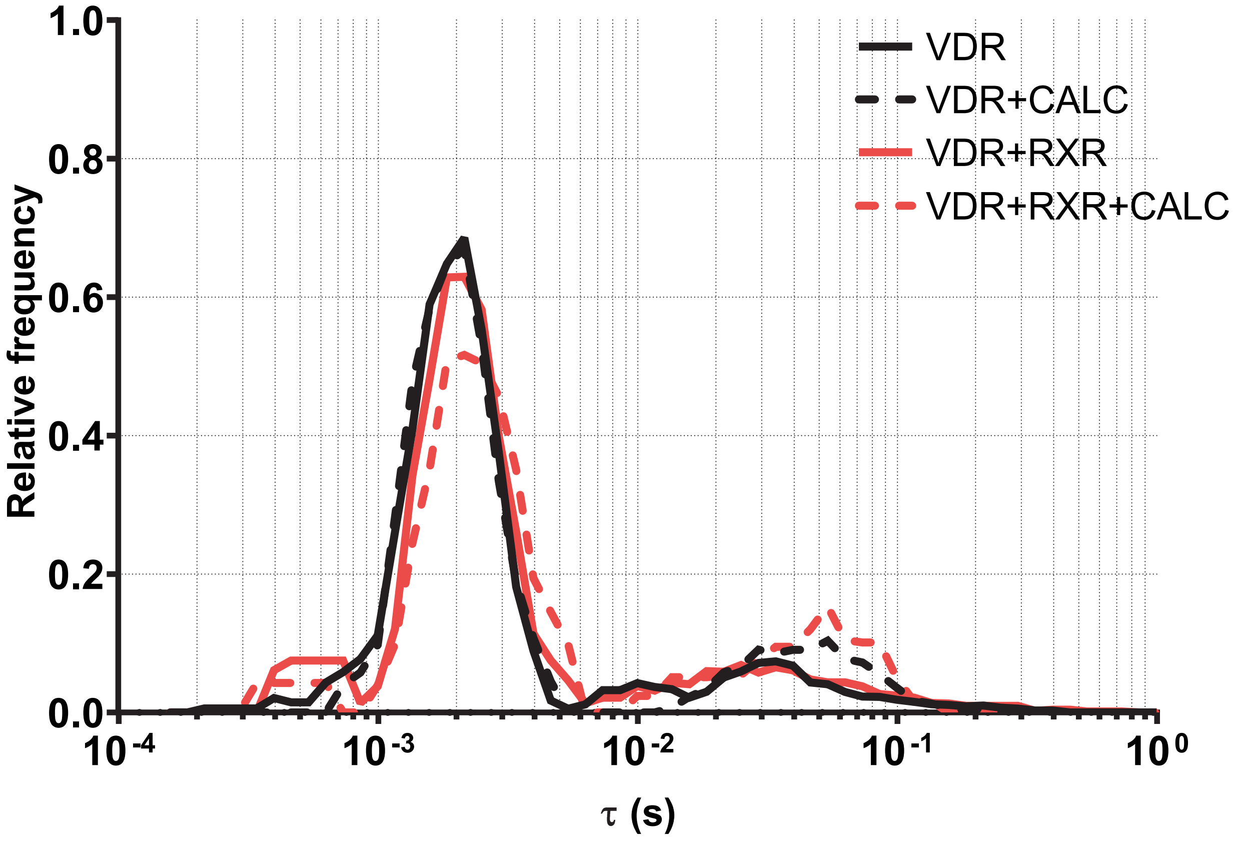

Fig. S3. Distribution of the diffusion times of the fast and slow components of EGFP-VDR derived from fits to a 2-component normal diffusion model also taking into account triplet state formation and blinking of the dye; areas under the curves indicate the fractions of the two components. Co-transfection of mCherry-RXR and treatment with VDR agonist (100 nM calcitriol) are indicated in the legend.

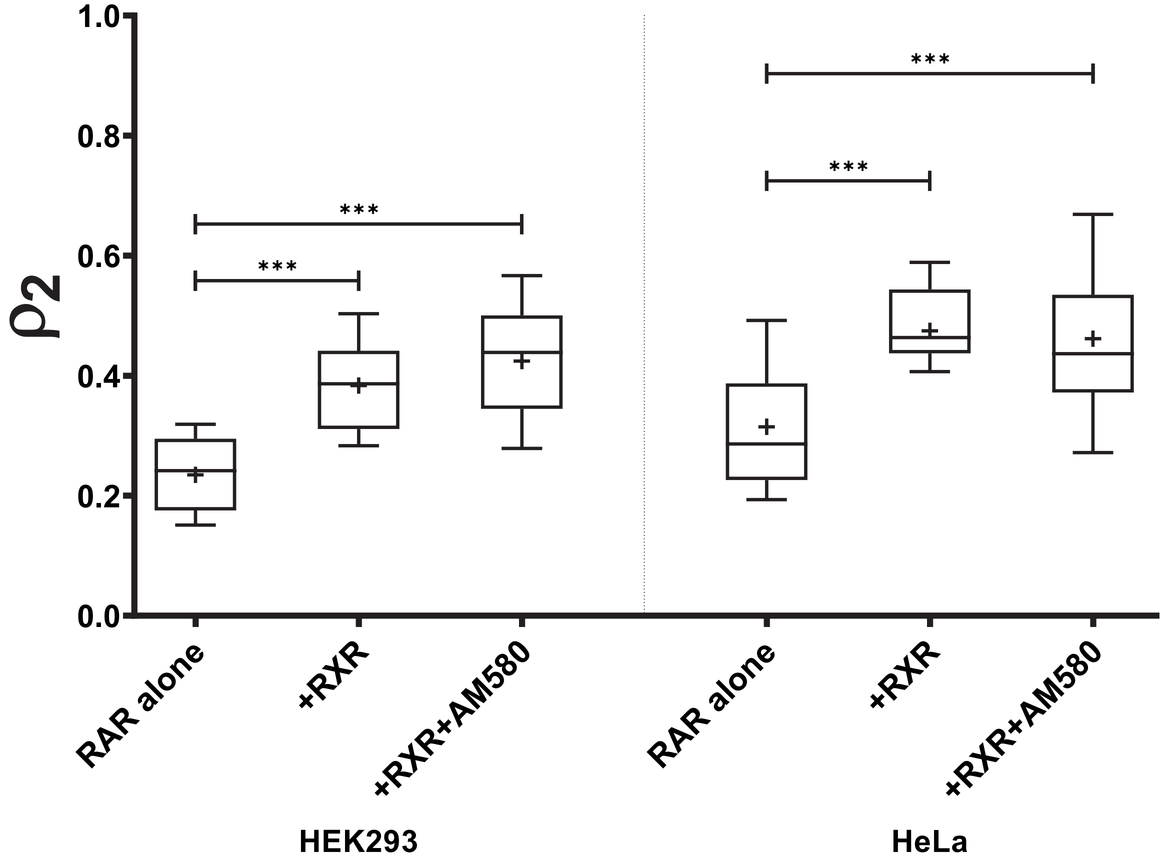

Fig. S4. Mobility parameters of EGFP-RAR expressed alone or co-transfected with mCherry-RXR in live HEK293 and HeLa cells with or without RAR agonist treatment. Boxes mark the 25th and 75th percentiles while whiskers the 10th and 90th percentile values. The horizontal line in the box represents the median. Averages are marked by "+". To compare averages, t-tests were performed; ***, $p < 0.001$.

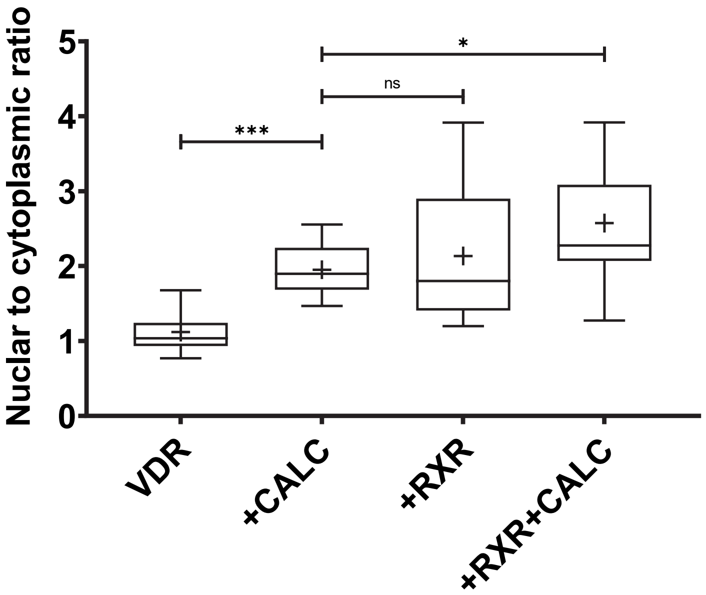

Fig. S5. Nuclear to cytoplasmic ratio of EGFP-VDR after calcitriol treatment (100 nM) and co-transfection of mCherry-RXR. Boxes mark the 25th and 75th percentiles while whiskers the 10th and 90th percentile values. The horizontal line in the box represents the median. Averages are marked by "+". To compare averages, t-tests were performed; *, $p < 0.05$; ***, $p < 0.001$; ns, not significant.

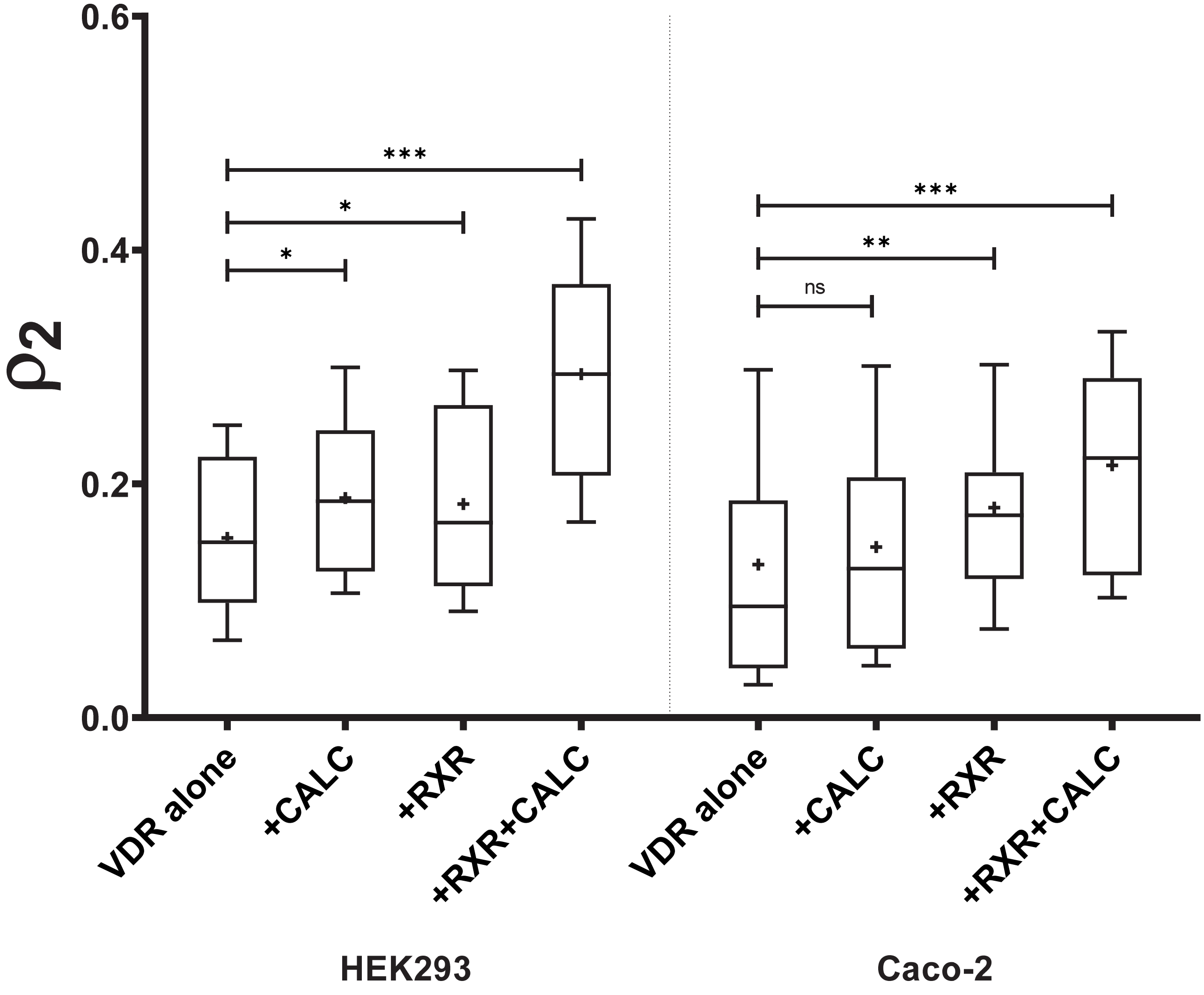

Fig. S6. Mobility parameters of EGFP-VDR expressed alone or co-transfected with mCherry-RXR in live HEK293 and Caco-2 cells with or without VDR agonist treatment. Boxes mark the 25th and 75th percentiles while whiskers the 10th and 90th percentile values. The horizontal line in the box represents the median. Averages are marked by "+". To compare averages, t-tests were performed; *, $p < 0.05$; **, $p < 0.01$; ***, $p < 0.001$; ns, not significant.

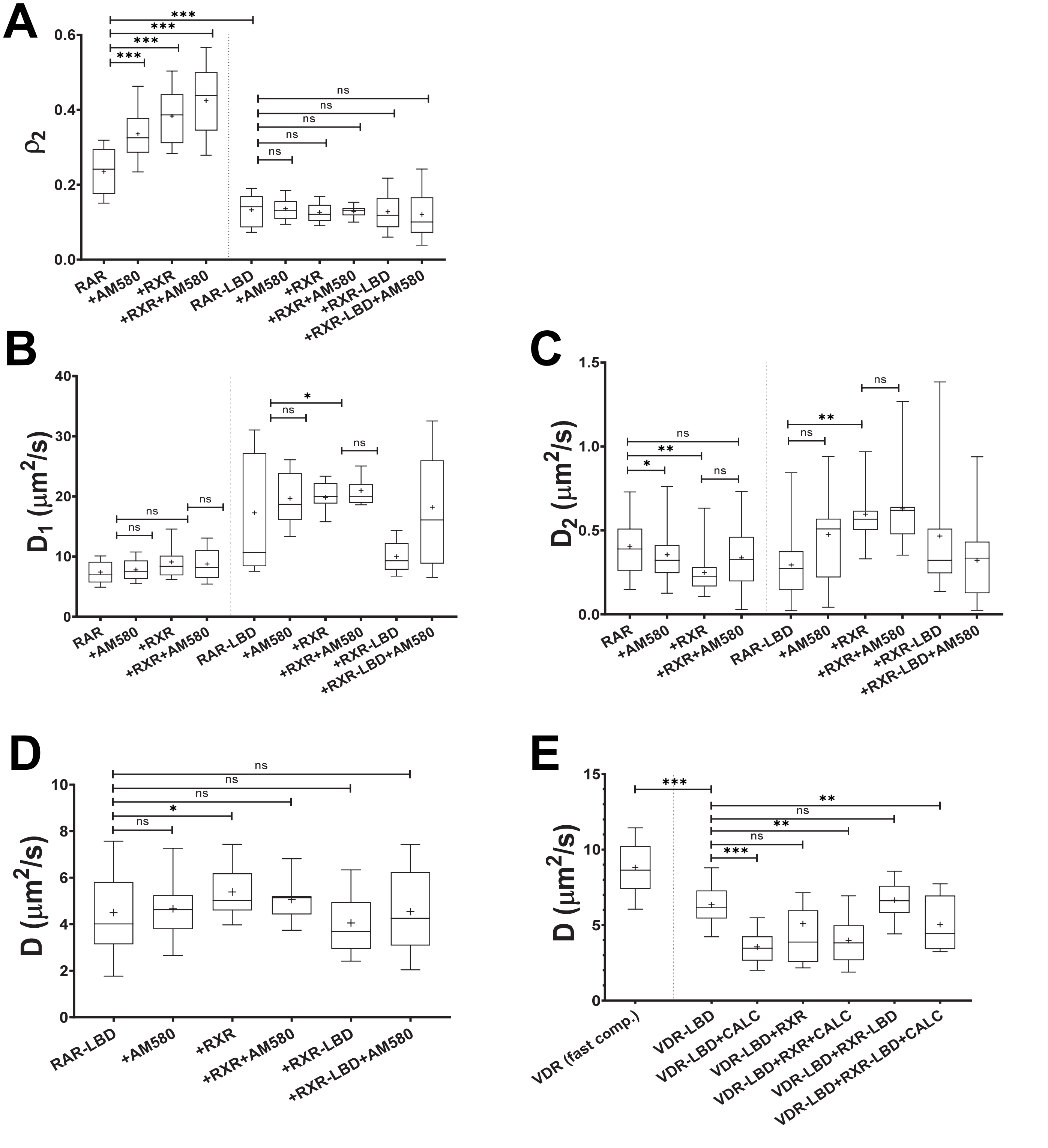

Fig. S7. Mobility parameters of EGFP-NR-LBDs lacking DNA-binding domains expressed alone or co-transfected with mCherry-RXR or mCherry-RXR-LBD in live HEK293 cells with or without agonist treatment. A) Fraction of the slow component of EGFP-RAR-LBD compared to EGFP-RAR. Cells were treated with 100 nM RAR agonist for 20 min (+AM580), and/or co-transfected with mCherry-RXR (+RXR) or its ligand binding domain mCherry-RXR-LBD (+RXR-LBD) lacking direct DNA-binding capacity. B) Diffusion coefficient of the EGFP-RAR-LBD fast component compared to EGFP-RAR. C) Diffusion coefficient of the EGFP-RAR-LBD slow component compared to EGFP-RAR. D) Diffusion coefficient of the EGFP-RAR-LBD when fitted with a 1-component normal diffusion model. E) Diffusion coefficient of the EGFP-VDR-LBD when fitted with 1 component compared to the fast component of EGFP-VDR.

Boxes mark the 25th and 75th percentiles while whiskers the 10th and 90th percentile values. The horizontal line in the box represents the median. Averages are marked by "+". To compare averages of ρ_2 or D from the one-component fits, t-tests were performed; the distributions of diffusion times weighted by their fractions were compared by an F-test. *, $p < 0.05$; **, $p < 0.01$; ***, $p < 0.001$; ns, not significant.

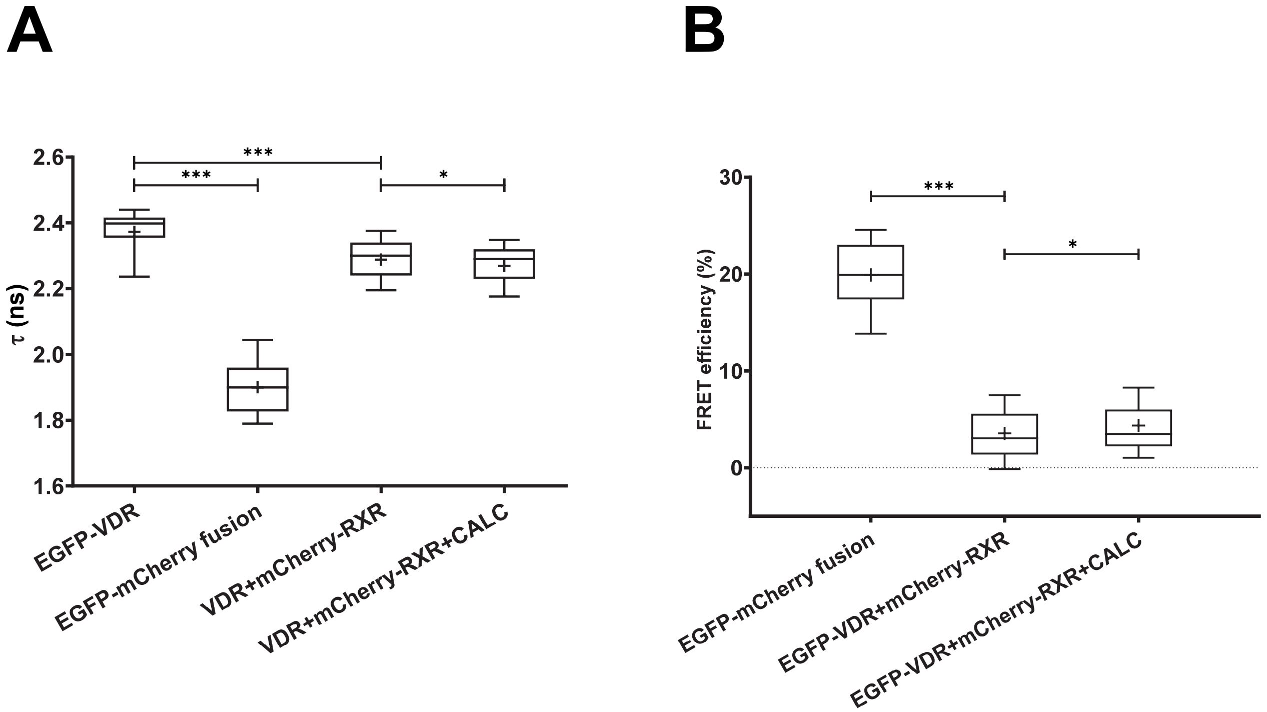

Fig. S8. FLIM-FRET measurements of heterodimerization between EGFP-VDR and mCherry-RXR.
A) Donor fluorescence lifetimes of the different samples. The fusion of EGFP and mCherry was used as a positive control. B) FRET efficiency measured between EGFP-VDR and mCherry-RXR in the absence or presence of calcitriol (100 nM), calculated by Eq. 9. Boxes mark the 25th and 75th while whiskers the 10th and 90th percentile values. The horizontal line in the box represents the median. Averages are marked by "+". To compare averages, t-tests were performed; *, $p < 0.05$; ***, $p < 0.001$.

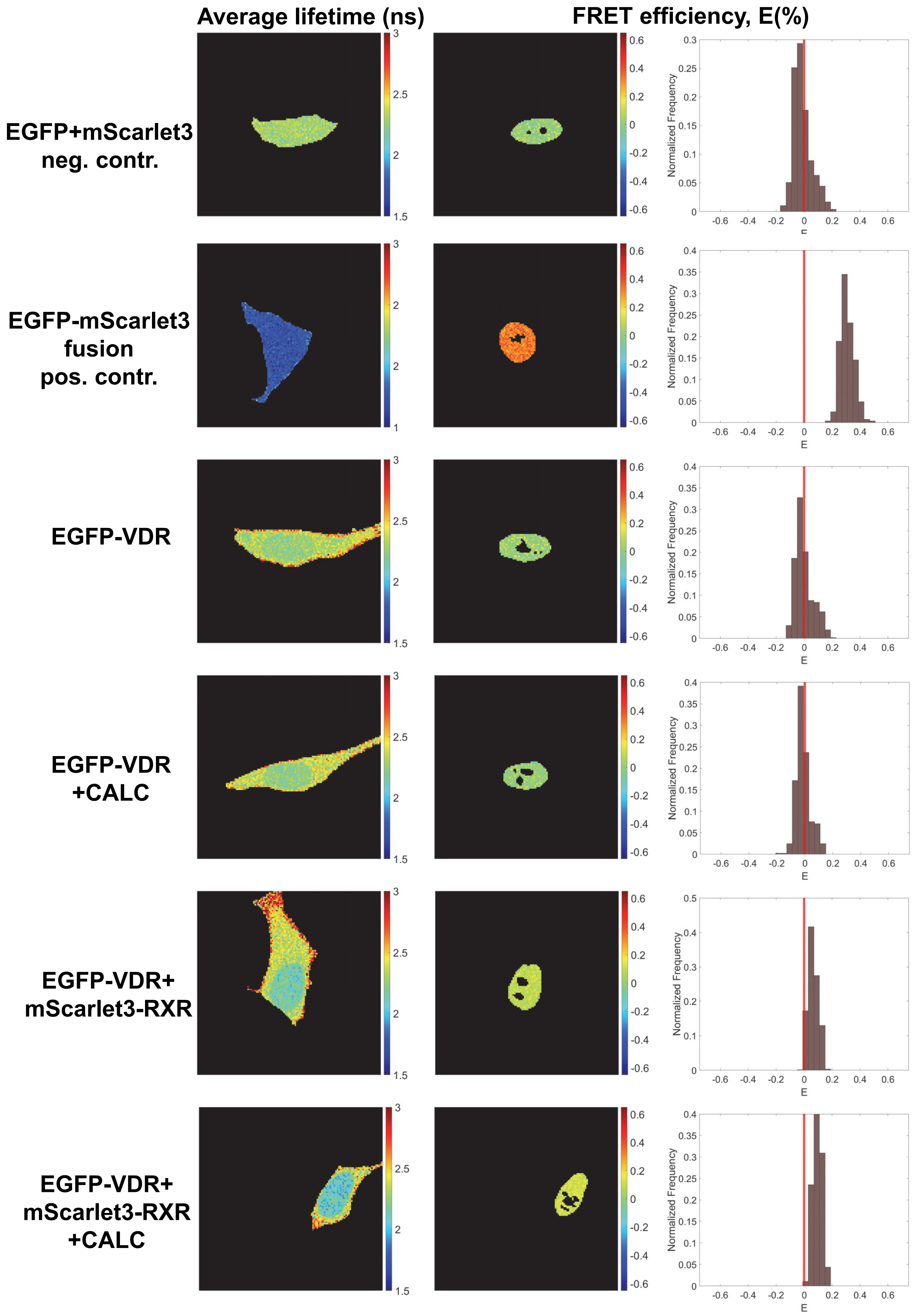

Fig. S9. Representative lifetime and FRET efficiency maps of a selected cell from different samples. On the average lifetime maps the whole cell is shown, while on the FRET efficiency maps the nucleus of the cell is indicated. FRET efficiency histograms display the pixelwise distribution of E values throughout the nuclei. Image size: 72x72 µm.

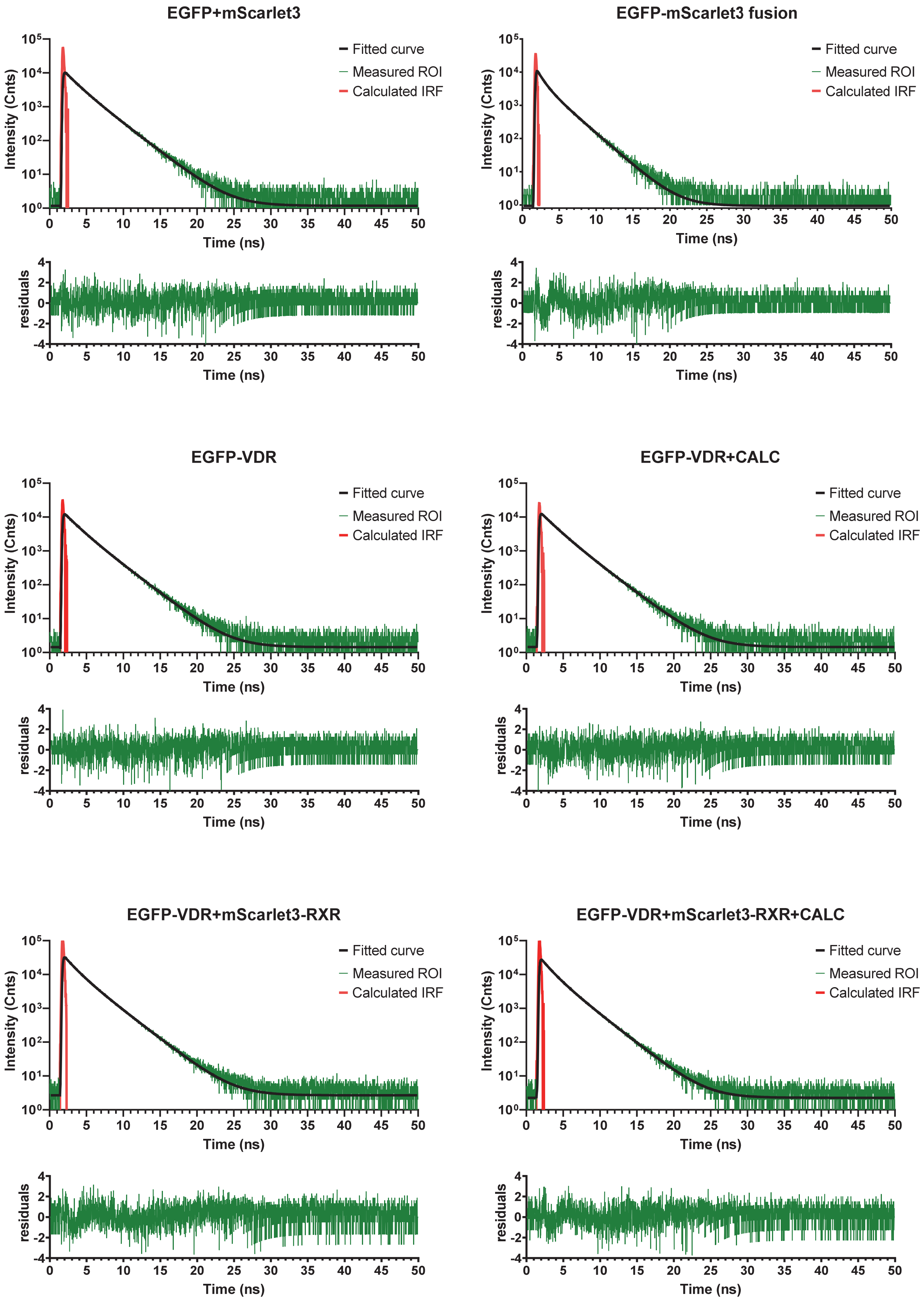

Fig. S10. Representative donor (EGFP) lifetime decay curves of different samples. Curves represent averaged fluorescence intensity decays of selected regions of interest (ROI) drawn in a cell nucleus. Curves were fitted with the SymphoTime 64 software to a multi-exponential reconvolution model with two lifetime components (see Eqs 7 and 8). Treatment with VDR agonist (100 nM calcitriol) is indicated in the headers.

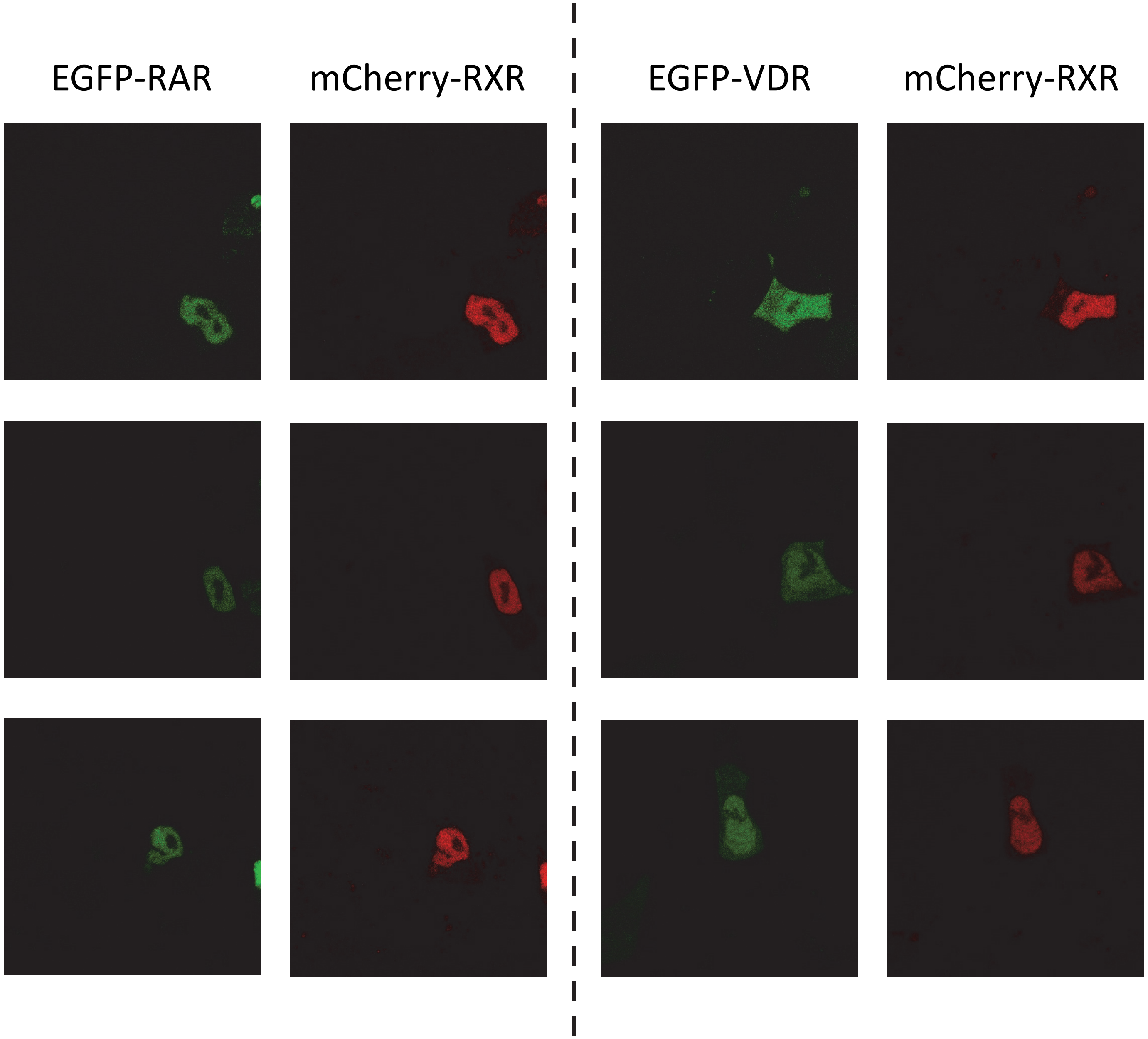

Fig. S11. Representative confocal images of EGFP-RAR/mCherry-RXR and EGFP-VDR/mCherry-RXR co-expressed in HEK 293 cells (image size: 71x71 µm).
